# Multimodal Structural Characterization of SARS-CoV-2 Spike Variants: Spectroscopic and Computational Insights

**DOI:** 10.3390/ijms262110342

**Published:** 2025-10-23

**Authors:** Tiziana Mancini, Nicole Luchetti, Salvatore Macis, Velia Minicozzi, Rosanna Mosetti, Alessandro Nucara, Stefano Lupi, Annalisa D’Arco

**Affiliations:** 1 Department of Physics, University La Sapienza, P.le A. Moro 2, 00185 Rome, Italy; salvatore.macis@uniroma1.it (S.M.); stefano.lupi@uniroma1.it (S.L.); annalisa.darco@uniroma1.it (A.D.); 2 Engineering Department, Università Campus Bio-Medico di Roma, Via Alvaro del Portillo 21, 00128 Rome, Italy; n.luchetti@unicampus.it; 3 Department of Physics, University of Rome Tor Vergata, Via della Ricerca Scientifica 1, 00133 Rome, Italy; velia.minicozzi@roma2.infn.it; 4 Department of Basic and Applied Sciences for Engineering (SBAI), Sapienza University of Rome, Via A. Scarpa 16, 00161 Rome, Italy; rosanna.mosetti@uniroma1.it

**Keywords:** SARS-CoV-2 variants, spike glycoproteins, ATR-IR spectroscopy, CD spectroscopy, MD simulations, secondary structure, hydrophilicity

## Abstract

The SARS-CoV-2 pandemic has driven the emergence of many viral variants carrying multiple mutations, particularly in the spike glycoprotein, which enhance viral adaptability and may alter the structure and functionality of the protein. Here, we present, to the best of our knowledge, the first systematic and comparative structural analysis of monomeric spike protein subunit 1 from three distinct SARS-CoV-2 variants at physiological pH (7.4). A multimodal approach was employed, integrating experimental techniques, including Attenuated Total Reflection Infrared and circular dichroism spectroscopies, with computational methods such as molecular dynamics simulations and surface polarity analyses. This combined approach allowed us to characterize the secondary structure composition, three-dimensional conformational organization, and solvent interaction profiles of each variant. Our findings reveal how the structural and functional properties of the spike protein subunit 1 are influenced by specific amino acid mutations. Indeed, the observed conformational changes and variations in solvent interactions have significant implications for viral infectivity and immune evasion. These findings contribute to the broader understanding of the evolution of SARS-CoV-2 variants and offer valuable insights for drug development, targeted prevention strategies, and biosensor design.

## 1. Introduction

The SARS-CoV-2 pandemic, which emerged in 2019, has been the largest-scale health emergency in recent centuries, causing more than 7 million deaths to date, as declared by the World Health Organization (WHO) [1,2]. The SARS-CoV-2 virus, responsible for this pandemic crisis, belongs to the Coronaviridae family, and is related to severe acute respiratory syndrome. Similarly to other Coronaviruses, SARS-CoV-2 shows a spherical enveloped structure (with a diameter between 80 and 120 nm) enclosing a single positive strand RNA. In addition to the different proteins included in the virus structure (envelope, membrane, nucleocapsid and spike proteins), the spike (S) glycoprotein plays the most important role in the viral transmission process. The S glycoprotein is the largest viral protein of the Coronavirus family, made up of 1273 amino acids and protruding from the viral surface, playing the key role in anchoring the virus to the human receptor ACE2 [3]. The S protein is composed of two subunits, S1 (residues 1–685) and S2 (residues 686–1273), each formed by different domains. In turn, the S1 subunit is composed of the N-terminal domain (NTD, residues 14–305) and the receptor binding domain (RBD, residues 319–541). The latter is the protein site that initially binds to ACE2, triggering the infectious process [4,5,6,7,8,9].

The circulation of the virus for almost a year resulted in a major step forward in its adaptation to humans [10,11]. Numerous mutations have accumulated in the viral RNA genome over time, giving rise to highly mutated forms of SARS-CoV-2 which have been recognized and classified into viral lineages known as Variants of Concern (VoCs) [12,13,14,15]. Some of these mutations led to changes in the amino acid sequences, such as single amino acid substitutions, amino acid deletions or insertions, and resulting in structural alterations of the S glycoproteins. Moreover, single-point mutations can even lead to marked functional changes, despite causing only minor alterations in the overall structure. Such mutations may affect local flexibility, disrupt or create hydrogen bonds, modify the electrostatic potential, or alter side-chain packing, which in turn can impact protein–protein interactions, stability, or receptor binding. These seemingly small perturbations can have long-range allosteric effects or shift the equilibrium between functional states, as shown by molecular dynamics studies on SARS-CoV-2 spike variants [16,17]. Many works on SARS-CoV-2 VoCs have documented the influence of mutations on S protein behavior, especially in terms of interaction bridges and strength with the ACE2 receptor, binding affinity [18,19,20,21], flexibility [22,23], and accessibility [14,24]. These findings point out possible differences and changes in protein properties, and as a consequence, how mutations affect the evolution and spread of the virus [6,18,22,23,24,25,26,27,28,29,30,31,32,33]. For instance, it is well known that the Omicron variant exhibits significantly improved transmissibility [34,35,36,37], with 50 mutations compared to the wild type (WT). Twenty-six of these mutations are unique to this variant and more than thirty concern the spike glycoprotein. Therefore, the success of each VoC relative to the previously dominant one was enabled by altered intrinsic functional properties of the virus and, to varying degrees, by changes to virus antigenicity, conferring the ability to evade a primed immune response [12,19,38,39]. In this context, the knowledge of the secondary structural characteristics of SARS-CoV-2 VoC proteins and their differences is of primary importance to understanding the connection with the spreading and infectious mechanisms.

Our work provides, to the best of our knowledge, a first systematic and comparative structural study of three distinct monomeric S1 subunits of SARS-CoV-2 VoCs, with the aim to investigate the presence of any potential difference in their 3D conformation and/or physic-chemical properties, which may be attributable to variations in their amino acid sequence. While the trimeric form is physiologically relevant, the monomeric one allows us to isolate the intrinsic structural effects of mutations for a systematic comparative study, without inter-protomer interactions or glycan shielding.

We employed a multimodal characterization approach, combining infrared (IR) and circular dichroism (CD) spectroscopies, well-established experimental methods for a non-invasive analysis of polypeptides and proteins [39,40,41,42,43,44,45,46,47], with molecular dynamics (MD) simulations and surface polarity analyses. Referring to them by their common names, we considered the Alpha variant (lineage B.1.1.7), Gamma variant (lineage P.1/P.1.1/P.1.2) and Omicron variant (lineage B.1.1.529).

The investigation of VoC mutations of the S1 protein structure is firstly provided by CD spectra in the wavelength range between 190 and 230 nm, providing information about their secondary structures, in terms of the α-helix, β-sheet, β-turn and random coil structures [42], and making CD spectroscopy an effective tool for providing a first visualization of structural changes resulting from viral mutations. A subsequent in-depth analysis of secondary structure has been performed by exploiting IR spectroscopy and focusing the study on the Amide I band (1590–1710 cm^−1^) [39,40,41]. IR and CD spectra are also strongly influenced by the overall three-dimensional (3D) conformational structure of the protein and, therefore, by its intrinsic conformational order, in terms of bond and angle symmetry [39,48,49,50], its packaging and folding, its hydrophilicity, and finally the strength of hydrogen bonds with the surrounding water [51,52]. The IR and CD spectral differences in Alpha, Gamma and Omicron S1 proteins are accompanied by the description of their behavior in water obtained through a computational approach employing ColabFOLD (v1.5.5: AlphaFold2 using MMseqs2, URL accessed on 21 November 2022), MD simulations and Protein-sol software (https://protein-sol.manchester.ac.uk/, accessed on 14 March 2025). Through this combined analysis, deep insight into the conformational structure of the three VoCs was obtained, pointing out important differences in their structural dynamics, from both experimental measurements and computational simulations. Our overall results represent a fundamental step in the non-invasive and non-destructive investigation and comparison of three dominant VoCs which affected Europe and the world during the period of 2020–2022. The knowledge of their secondary structure and conformational behavior in water sheds light on their functionalities and the structural dynamics underlying the infectious mechanism. Therefore, these results may have implications for several applicative fields, including the design of innovative optical biosensors, where viral proteins may be used as a potential biomarker [53,54,55,56,57,58,59], as well as drug design, preventive action development and further structural studies.

## 2. Results

**Figure 1:**
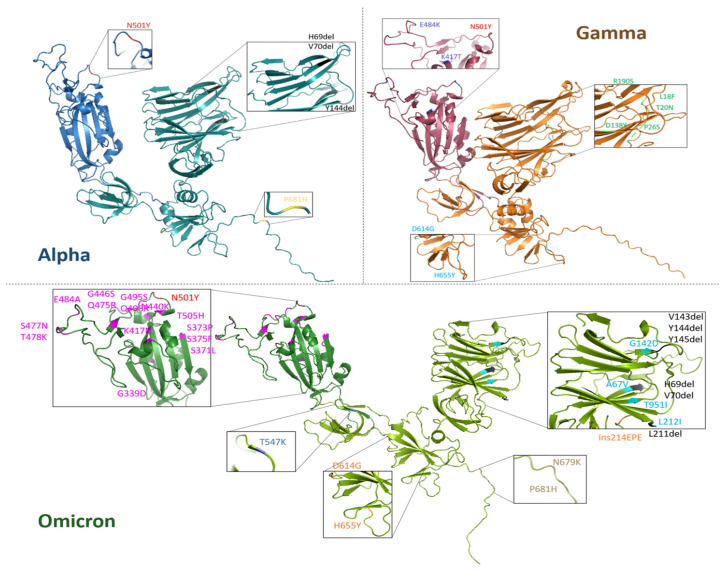
Three-dimensional visualization of monomeric S1 protein variants. Alpha (blue), Gamma (orange) and Omicron (green) SARS-CoV-2 variants. For each S1 protein, the RBD region (residues 319–541) is highlighted with a darker color with respect to the whole protein. For each variant S1 protein, respective mutations are zoomed in on and listed in the inset. The common mutation N501Y is indicated with the same color (red) and the deletions are marked in black.

Monomeric S1 proteins from Alpha, Gamma and Omicron SARS-CoV-2 variants have been modeled with the AlphaFold2 algorithm, outperformed by ColabFOLD (v1.5.5: AlphaFold2 using MMseqs2, URL accessed on 21 November 2022) [59], starting from their amino acid sequences, provided by Sino Biological Europe GmbH, and reported in the Supplementary Information (SI) (paragraph S1). In Figure 1, three-dimensional models of the three S1 proteins are visualized with PyMOL (ver. 3.0.5) and the names and positions of each mutation are pointed out. RBDs are highlighted in a darker color for each variant protein.

### 2.1. CD Spectral Analysis

**Figure 2:**
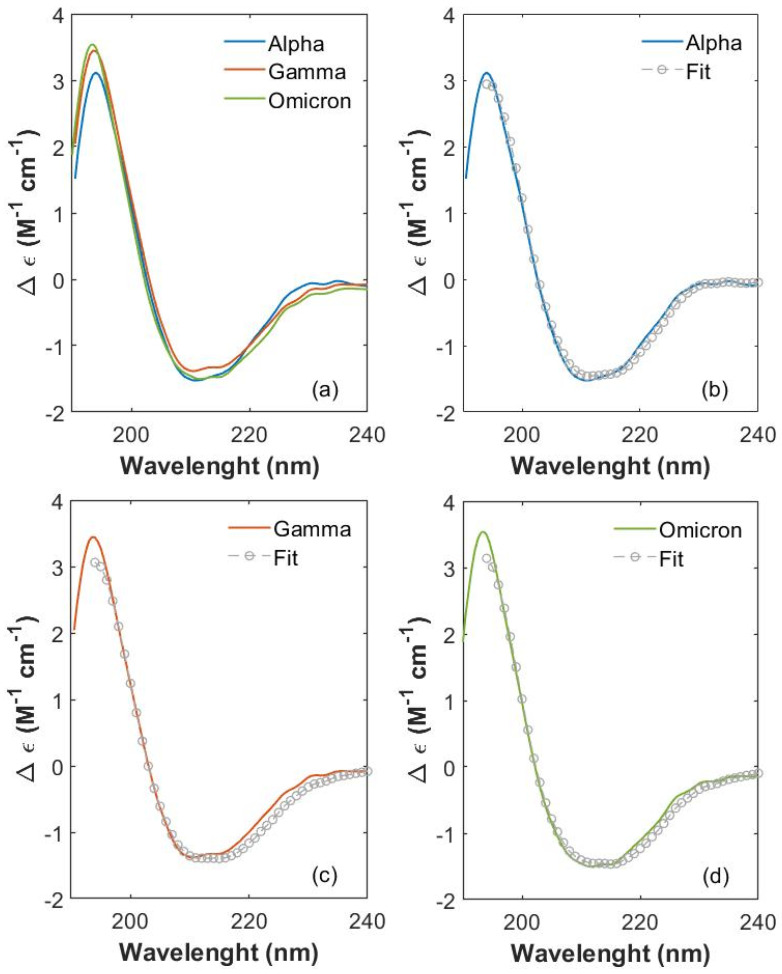
Comparison of S1 CD spectra between 190 and 230 nm. (**a**) Comparison of CD spectra between 190 and 230 nm of S1 proteins from SARS-CoV-2 Alpha (blue), Gamma (orange) and Omicron (green) variants. Single variant CD spectra (colored curves) superimposed to fitting curves (gray open circles), for (**b**) Alpha, (**c**) Gamma and (**d**) Omicron proteins.

CD spectra (expressed in terms of ellipticity variation Δε) [42] were collected for the S1 proteins of SARS-CoV-2 variants (Alpha, Gamma and Omicron) between 190 and 230 nm. In this spectral region, the protein absorption is mainly due to the peptide bond with its n-π* transition (around 210 nm) and π-π* transition (around 190 nm) [42,43,44]. CD spectra (colored lines) are shown in Figure 2a for Alpha, Gamma and Omicron S1 proteins. Noticeable differences occur in the CD spectra of the three S1 protein variants, proving that mutated proteins are structurally different from each other.

A spectral variation is noticeable at the lowest wavelengths (190 nm), where CD absorptions of Gamma and Omicron S1 proteins have a more intense and broader peak (centered at about 194 nm) compared to the Alpha one. In this wavelength range, both the positive dichroic absorption of *β*-sheet structures and the negative absorption of *β*-turn structures give their competitive contribution [42].

An evident variation is also visible between 200 and 220 nm, where the negative absorption contribution is due to both β-sheets and α-helix structures. In particular, the chirality of α-helices gives rise to the characteristic modulation, which appears as two negative and partially overlapping peaks [42]. These peaks are clearly distinguishable for each spectrum, located at 211 nm and 215 nm for the Alpha S1 protein, at 210.5 nm and 215 nm for Gamma S1 and at 212 nm and 215 nm for Omicron S1. The variations of *β*-sheets’ and *α*-helices’ contributions in Alpha, Gamma and Omicron proteins appear also at the edge of the negative dip between 218 and 240 nm, where the three CD spectra clearly show different behaviors. In particular, in this frequency interval, the CD spectrum of the S1 Omicron protein (green curve) presents lower values of Δε (calculated as reported in S2) compared to Alpha and Gamma CD spectra (blue and orange curve in Figure 2a, respectively). It is worth mentioning that the WT S1 protein has been measured, too, in order to observe the extent of structural change starting from the first virus and going on as long as mutations occur. All four of the CD spectra are comparatively reported in SI (see S3, Figure S1). Significant spectral variations are visible at the low wavelengths (190 nm) and at higher wavelengths (between 200 and 230 nm), testifying to an important effect of mutations on variant’s protein structure. CDPro software (https://sites.google.com/view/sreerama, accessed on 8 September 2025) was used to perform the CD spectra deconvolution of the three proteins from 195 to 230 nm. We selected this wavelength range because it is the largest common interval where all the spectral databases employed for the CD analysis efficiently work. Final fitting curves are displayed in Figure 2b, Figure 2c and Figure 2d (gray circles) for Alpha, Gamma and Omicron S1 protein spectra, respectively, as explained in the Materials and Methods Section. From the deconvoluted analysis, the percentage content of the secondary structure was deduced and is reported in Table 1 for each variant S1 protein as the MEAN ± standard deviation (SD) calculated over the results obtained from the three algorithms and the six reference basis sets. See details for analysis in the Materials and Methods Section.

### 2.2. IR Spectral Analysis

**Figure 3:**
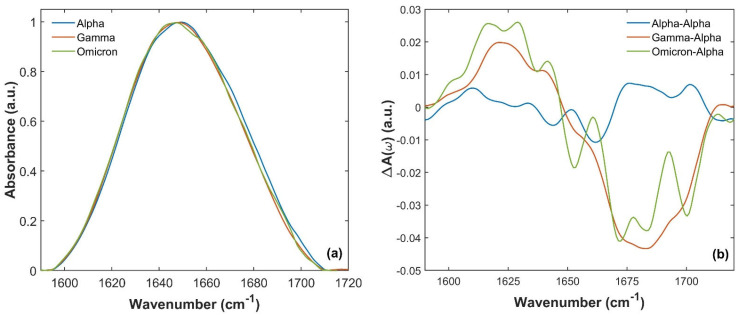
Comparison of Amide I S1 absorption spectra for the three variants between 1590 and 1720 cm^−1^. (**a**) Comparison of the S1 absorption spectra of Alpha (blue curve), Gamma (orange curve) and Omicron (green curve) variants of the SARS-CoV-2 virus. (**b**) A(ω)_(Gamma)_-A(ω)_(Alpha)_ (orange line) and A(ω)_(Omicron)_-A(ω)_(Alpha)_ (green line) differences compared to the reproducibility of the SARS-CoV-2 S1 Alpha absorption spectrum. This reproducibility was estimated by the difference in A(ω)_(Alpha)_-A(ω)_(Alpha)_ measured in two different measurement runs (blue curve). A sizeable difference (well beyond the reproducibility of the individual absorption spectra) was observed when comparing the absorption of Gamma and Omicron with the Alpha one.

Figure 3a shows the comparison of Amide I absorption bands from 1590 to 1720 cm^−1^ of Alpha (blue curve), Gamma (orange curve) and Omicron (green curve) SARS-CoV-2 variants. Slight differences can be recognized in the absorption band shape and spectral maxima frequency position. In particular, looking at Figure 3a, a slight shift in Amide I absorption maxima occurs when comparing the spectra of Alpha, Gamma and Omicron S1 proteins. The maximum is located at (1650 ± 1) cm^−1^ for the Alpha Amide I absorption band, the same as for the WT S1 protein, as reported in previous studies [60,61]. In contrast, the Gamma and Omicron Amide I absorption bands have maxima located at (1648 ± 1) cm^−1^ and (1647 ± 1) cm^−1^, respectively.

These differences are further highlighted by calculating the differences in A(ω)_(Gamma)_-A(ω)_(Alpha)_ and A(ω)_(Omicron)_-A(ω)_(Alpha)_ (orange and green lines in Figure 3b, respectively) and comparing them, for instance, with the reproducibility of the absorption measurements [60,61], estimated by the difference between A(ω)_(Alpha)_-A(ω)_(Alpha)_ for two different measurement runs (blue line in Figure 3b). A sizable difference was observed well above the reproducibility of the absorption spectra measurements, which is approximately ~1%. In particular, both Gamma and Omicron SARS-CoV-2 S1 have a lower absorption intensity between 1665 and 1700 cm^−1^ (in agreement with the differences observed in Figure 3a), and a slightly more intense signal at lower frequencies.

**Figure 4:**
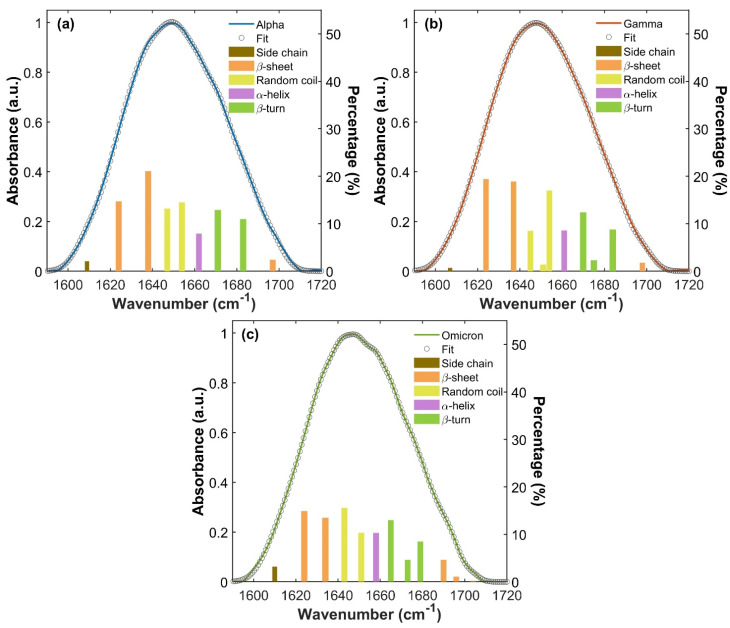
Amide I absorption bands of S1 proteins for the three variants. IR Amide I band of Alpha (**a**), Gamma (**b**) and Omicron (**c**) S1 proteins and their global fitting (gray circles curve), referring to the left y-axis. For each Amide band, the deconvolution into Gaussian components is represented through the underlying bars, with assignments described through their colors (as explained in the legend). Their heights show the percentage contribution of each spectral component (right y-axis).

These spectral differences correspond to a different distribution of spectral absorption components, in terms of shifts in frequencies and/or variations in absorption intensity and therefore reflect different IR vibrations of proteins’ intrinsic structures. To better understand this behavior, Amide I absorption bands of S1 monomeric variant proteins were deconvoluted into Gaussian components using a multi-Gaussian fitting approach (as described in the Materials and Methods Section). The results are displayed in Figure 4a, Figure 4b and Figure 4c for Alpha, Gamma and Omicron, respectively. In each panel, the convoluted bands are represented by underlying bars, whose height corresponds to the percentage contribution of each spectral component to the overall Amide I band intensity. The assignment to a specific secondary structure is indicated by different colors (orange for *β*-sheet, yellow for random coil, purple for *α*-helix, green for *β*-turn and brown for side chain contribution). Table S1 in S4 summarizes Amide I vibrational frequencies for each S1 variant protein and their assignment to secondary structures. Notably, the Amide I band deconvolution of S1 protein from the WT has already been studied and reported in our previous works [60,61].

For all three S1 proteins, *β*-sheet vibrations (orange bars in Figure 4a–c) arise with two different contributions (see S4, Table S1), one at a low frequency attributable to *ν*_⟂_ mode, and the other at a high frequency attributable to *ν*_//_ mode [39,40,41,60,61]. This doublet absorption is generally associated with an antiparallel *β*-sheet structure [39,40,41,60,61,62]. In particular, both Alpha and Gamma S1 proteins show two low-frequency *β*-sheet *ν*_⟂_ absorptions around 1624 and 1637 cm^−1^ and one high-frequency *β*-sheet *ν*_//_ absorption at about 1697 cm^−1^. The Omicron S1 protein exhibits two *β*-sheet *ν*⟂ absorption peaks lying at 1624 and 1634 cm^−1^, slightly lower in frequency compared to Alpha and Gamma proteins’ *β*-sheet *ν*_⟂_ vibration modes and with an evident lower percentage contribution. At a high frequency, the Omicron Amide I band shows three *β*-sheet *ν*_//_ bands around 1690, 1696 and 1704 cm^−1^, providing a greater contribution compared to Alpha and Gamma.

In the Alpha, Gamma and Omicron S1 protein, the *α*-helix vibrational mode (purple bar in Figure 4a–c) rises as a single absorption peak. It lies around 1661 cm^−1^ for Alpha and Gamma S1 proteins, while it is slightly redshifted in the Omicron S1 protein located at 1658 cm^−1^.

Random coil (yellow bars in Figure 4a–c) structure vibrations are found between 1643 cm^−1^ and 1654 cm^−1^. In the Alpha protein Amide I band, they rise at 1647 and 1654 cm^−1^, while in the Gamma protein they are found at 1645 and 1654 cm^−1^, together with a weak shoulder at 1651 cm^−1^. In the Omicron S1 protein, random coil vibrations are slightly redshifted, lying at 1643 and 1651 cm^−1^. Finally, *β*-turn (green bars in Figure 4a–c) contributions are found between 1665 cm^−1^ and 1684 cm^−1^. They rise as two absorption bands at 1671 and 1683 cm^−1^ in the Alpha S1 protein, while three *β*-turn peaks are found at 1670, 1675 and 1684 cm^−1^ in the Gamma Amide I band. In the Omicron protein Amide I band, *β*-turn structures rise with three absorptions placed at 1665, 1673 and 1679 cm^−1^, again shifted to lower frequencies compared to the Alpha and Gamma Amide bands.

The percentage content of each secondary structure was estimated by calculating the ratio of the integrated intensity of its spectral components to the total integrated intensity of the Amide I band [60,61,62,63,64,65], after the subtraction of side chain vibrations from the total area [61]. Results are reported in Table 2 for each variant, expressed as the average and the SD of the estimation of each secondary structure content across different protein depositions, as explained in the Materials and Methods Section.

### 2.3. AlphaFold2 Prediction and Molecular Dynamics Simulations

S1 proteins of Alpha, Gamma and Omicron variants were modeled using AlphaFold2, starting from their amino acid sequences (see S1), provided by Sino Biological Europe GmbH (Eschborn, Germany). According to AlphaFold2 predictions, the S1 protein appears to assume two possible different configurations. In the first, the protein is stabilized in a “closed” state, with the RBD and NTD domains placed very close to each other and somehow providing a compact and folded conformation. In the second, S1 is stabilized in an “open” state, with the RBD and NTD domains placed further from each other compared to the “closed” state (see S5, Figure S2). The distinction between the “open” and the “closed” state is based on the distance between the centers of mass (COM) of NTD and RBD, which are reported in Table S2, paragraph S5. It appears that, for each variant, the “closed” state has an NTD-RBD distance that is lower than 5 nm, while the “open” configurations show an NTD-RBD distance that is larger than 5 nm. Following the approach of de Souza et al. [16,17], we also computed the RBD opening angle, θ, to classify “open” and “closed” conformations. Specifically, θ was defined as the angle between (i) the vector connecting the COM of the Cα atoms of residues 1–270 and the COM of the Cα atoms of residues 571–574 and 301–305, and (ii) the vector connecting this latter COM to the COM of the Cα atoms of residues 315–500 (Figure S3). In Table S3 we report the values of θ for the initial structures and as averages over the last 100 ns of the MD simulations. Consistent with de Souza et al. [16,17], we classified conformations as “open” when θ > 80°. We note that the classification based on θ is consistent with the one obtained using the NTD–RBD distance criterion, further supporting the robustness of our definition of open and closed states.

In order to predict and simulate how the three different proteins behave and adapt in an aqueous environment under the serological condition pH of 7.4, MD simulations have been performed via the GROMACS v. 2022.3 package [66,67] on Alpha, Gamma and Omicron protein models. To evaluate the goodness of the predictions, the Local Distance Difference Test (plDDT) was performed for all three variants, observing that both “open” and “closed” states present a similar plDDT value. Therefore, MD simulations were performed starting from both the “open” and the “closed” states for each variant. The CHARMM22* force field was applied, and the proteins were allowed to evolve for 600 ns, a time that is sufficient for a reasonable numerical convergence [68,69]. This was evaluated by observing the Root Mean Square Deviation (RMSD) values of atomic positions throughout the simulation, as reported in the SI (see S6, Table S4 and Figure S4). The time-dependent behavior of the Radius of Gyration (R_g_) was studied for both closed and open models for all three S1 proteins (see S6, Figure S5). For Alpha, Gamma and Omicron, closed models retained a quite stable R_g_ value and preserved the closed configuration for the whole time. In the case of the initial open models of Alpha and Gamma, these tended to rearrange over time, decreasing their R_g_ values until reaching a closed state. On the other hand, the initial open model of Omicron maintained a stable R_g_ value throughout the simulation, remaining in an open state with a large R_g_ value. Initial and final R_g_ values for both “open” and “closed” states were calculated as the average over the first 50 ns and the last 100 ns of simulation, respectively. The results are reported in Table 3, while R_g_ curves for all six models are provided in the SI (see S6, Figure S5).

**Figure 5:**
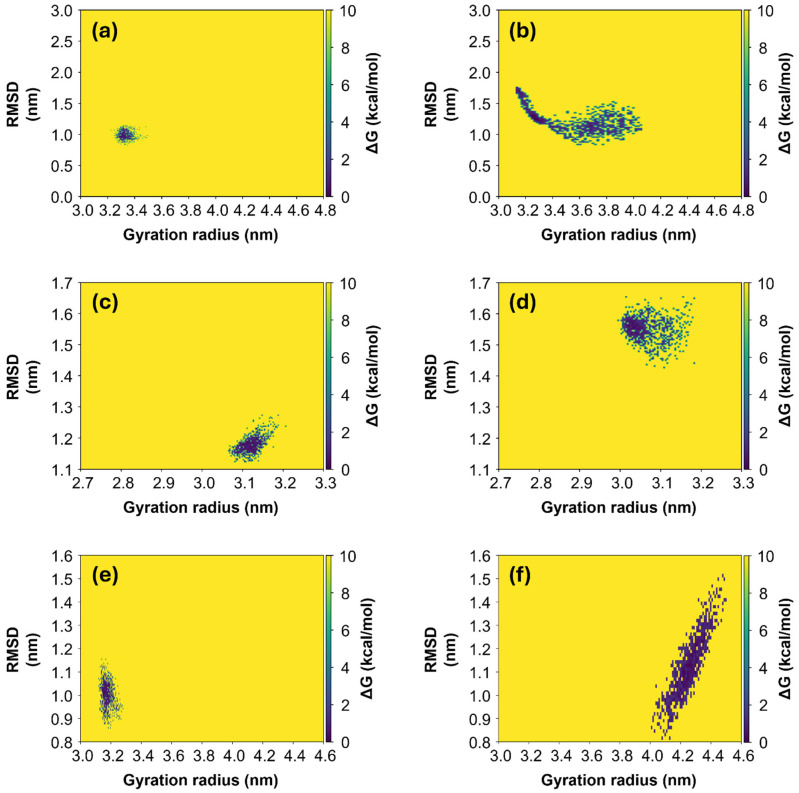
FES maps for the three variants of S1 protein. FES maps of Alpha (**a**,**b**), Gamma (**c**,**d**) and Omicron (**e**,**f**) are computed over the last 100 ns of each MD simulation, both for the systems starting from the initial “closed” state (left panels) and from the “open” state (right panels). The data are represented in the phase [R_g_; RMSD], where each point represents the protein configuration recorded with a dt = 0.1 ns.

As expected, the final R_g_ values, calculated as the average over the last 100 ns of simulation, correspond to the most stable configuration, with the lowest value of Free Energy Surface (FES). FES values have been calculated and two-dimensional heatmaps were computed as a function of RMSD and R_g_ values. They are shown in Figure 5 for Alpha, Gamma and Omicron variants, respectively, for both initial “closed” (left panels) and “open” states (right panels). Generally, it can be observed that for all three variants, the systems derived from the initial “closed” state (left panels in Figure 5) appear to be more stable in comparison with the ones derived from the initial “open” state (right panels in Figure 5). In fact, in the first case, the diffusion area of points in the phase space [R_g_; RMSD] is smaller with respect to the diffusion area in the second case, meaning that the protein needs to explore a small range of R_g_ values in order to achieve the equilibrium condition. In contrast, proteins derived from the initial “open” state need to span a larger space before finding the most stable configuration. In particular, the Omicron S1 protein derived from the initial “open” state (Figure 5f) is the only one exploring a range of larger R_g_ values, from 4.0 to 4.5 nm, and here it finds an equilibrium condition, differently from the other protein models which assume only smaller R_g_ values throughout the whole simulations. Root Mean Square Fluctuations (RMSFs) of the Cα atoms for the three variants (see S6, Figure S6) are larger for the “open” Alpha variant, consistent with its broader range of Rg values (Figure 5b).

Computational results for the percentage contents of secondary structures for both the “open” and “closed” states of the three variants are reported in Table 4. The results are calculated as the average and SD of each secondary structure content over the last 100 ns of the simulation. We notice that these values are essentially identical between the “open” and “closed” conformations, with differences falling within the statistical fluctuations of the simulations. Therefore, the observed secondary structure is robust and independent of the starting conformation.

### 2.4. Hydrophilic Calculation and Surface Polarity Computation

Hydrophilicity properties of the three VoC S1 proteins were evaluated, first computing their Gravy value using the ProtParam web-server (https://web.expasy.org/protparam/, accessed on 12 February 2025). The closer the Gravy value is to zero, the greater is the protein hydrophobicity. The results show that the Alpha and Omicron S1 proteins have the same Gravy value of −0.268, while the Gamma S1 protein has a Gravy value of −0.246.

**Figure 6:**
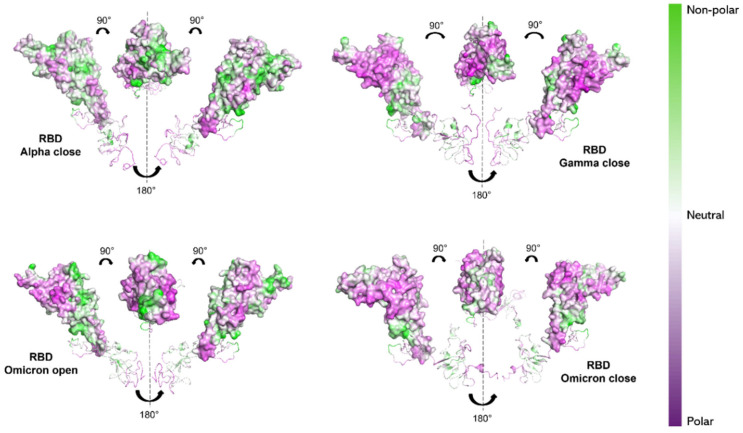
NPP surface ratio of the RBDs for the three variants. NPP ratio distribution is computed for RBDs’ surface for Alpha (closed state), Gamma (closed state) and Omicron (open and closed state). The color scale is shown on the right: green, white and purple colors correspond to hydrophobic, neutral and hydrophilic behavior, respectively.

This kind of calculation depends solely on the hydropathy of the individual amino acids, and therefore only on the protein’s primary structure. To verify how the 3D conformation of the three S1 proteins influences their hydrophilic properties and interaction with the solvent, non-polar to polar (NPP) ratio surfaces [70,71] were computed using Protein-sol software (https://protein-sol.manchester.ac.uk, accessed on 14 March 2025). The results are presented for S1 proteins of Alpha, Gamma and Omicron variants in the final configuration (600 ns), as provided by MD simulation. In particular, RBD and NTD are highlighted, as these are the regions where the majority of mutations occur (see Figure 1). For the Alpha and Gamma variants, only the “closed” states’ surfaces are shown, as both their initial “open” and “closed” states reached a “closed” configuration by 600 ns. For the Omicron S1 protein instead, both the “open” and “closed” states are shown. Noticeable differences can be observed in the NPP ratio behavior of RBDs. Alpha variant RBD shows a predominantly neutral/hydrophobic behavior (white/green region in Figure 6), in contrast to the Gamma and Omicron RBDs (both in “open” and “closed” states), which reveal strongly hydrophilic areas (purple regions Figure 6).

**Figure 7:**
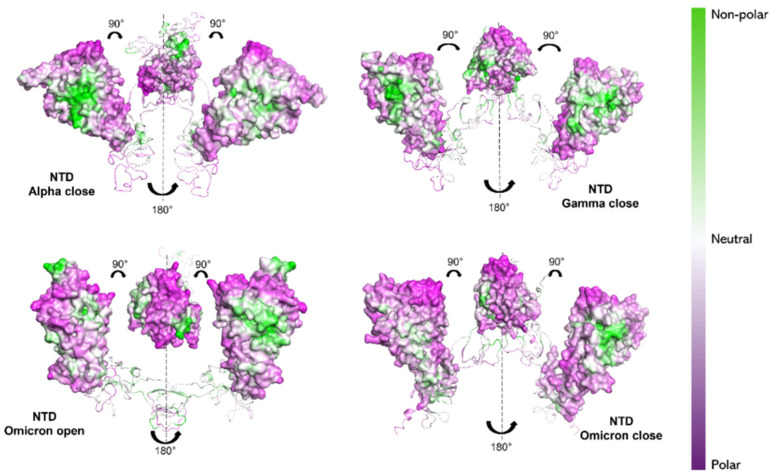
NPP surface ratio of the NTDs for the three variants. NPP ratio distribution is computed for NTDs’ surface for the Alpha S1 protein (closed state), the Gamma S1 protein (closed state) and the Omicron S1 protein (open and closed state). The color scale is shown on the right: green, white and purple colors correspond to hydrophobic, neutral and hydrophilic behavior, respectively.

Observing the NTDs, the Alpha variant still shows a more hydrophobic character (white/green regions in Figure 7) compared to the other two variants. In particular, Omicron (both in “open” and “closed” states) exhibits the most hydrophilic NTD character (purple regions in Figure 7), compared to Gamma and Alpha NTDs.

## 3. Discussion

SARS-CoV-2 VoCs emerged as a result of multiple mutations in the genomic sequence of the SARS-CoV-2 virus during the pandemic evolution, leading to structural differences in both their spike and nucleocapsid proteins. It is known that these changes influence protein behavior, especially in terms of interaction bridges and strength with the ACE2 human receptor, binding affinity [18,19,20,21], flexibility [22,23], and accessibility [14,24]. The study of mutations reveals that not all domains of the S protein are equally susceptible to mutations. The S1 subunit, the one that actively anchors the ACE2 receptor through the RBD domain, harbors most mutations, compared to the S2 subunit. In particular, NTD and RBD, located in S1 subunits, are the regions presenting the most important variations [14].

Here, we discuss and compare experimental IR and CD data, as well as computational results, to detect differences induced by amino acid sequence mutations in the S1 subunit of Alpha, Gamma and Omicron VoCs of the SARS-CoV-2 virus.

The evidence of structural changes immediately results in CD spectra from recognizable variations at low wavelengths, where S1 from Gamma and Omicron variants exhibit a more intense and broader peak around 194 nm compared to the Alpha variant. This is due to the different content of CD-active amino acids in each subunit, such as Alanine, Valine, Leucine and Asparagine [72] and the competitive contribution of *β*-sheet and *β*-turn structures.

Significant differences in the CD spectra also occur between 200 and 240 nm: here, protein dichroism is generally very responsive to the abundance of *α*-helix, *β*-sheet and coil structures [42,45,73]. These changes in CD spectra recorded for the S1 proteins of three VoCs are resulted to be the effect of mutations accumulating in the VoCs’ amino acid sequences and of the subsequent adaptations of their secondary structure.

To further shed light on possible structural modifications occurring in S1 proteins due to their mutations, the IR Amide I absorption band was inspected, revealing significant differences in the three variants (Figure 3 and Figure 4).

Indeed, IR deconvolution analysis highlights different shapes and distributions of Amide I spectral components, due to different vibrations in proteins’ structures. Among the most evident variations, *β*-sheet *ν*// modes occur as three distinct components in the Omicron Amide I band (see Figure 4c and see S4, Table S1), accounting for 6% of the whole protein secondary structure. In contrast, Amide I spectra of Alpha (Figure 4a) and Gamma (Figure 4b) variants show one single *β*-sheet *ν*// component, corresponding to about 2% of the total intensity. On the other hand, in all three VoCs, *β*-sheet *ν*⟂ vibrations occur with two intense peaks at low frequencies, but they contribute with a higher integrated intensity in Alpha and Gamma (between 36% and 38%), while they only constitute a lower percentage in Omicron absorption (around 28%).

For what concerns the *α*-helix absorption band, in the Omicron S1 protein it constitutes about 10%, while in Alpha and Gamma it is about 8%. Finally, slight changes concern *β*-turn absorptions, occurring as two peaks in Alpha Amide bands and as three distinct peaks in Gamma and Omicron.

The influence of mutations on S protein secondary structure has been just slightly studied [19,22,28], mostly by employing computational methods, which assume that mutations cause small changes in the 3D distribution, conformation and flexibility of protein structures.

In Table 1, Table 2 and Table 4 we provide the secondary structures’ contents of each protein variant obtained through CD and IR spectroscopy data and MD simulations.

**Figure 8:**
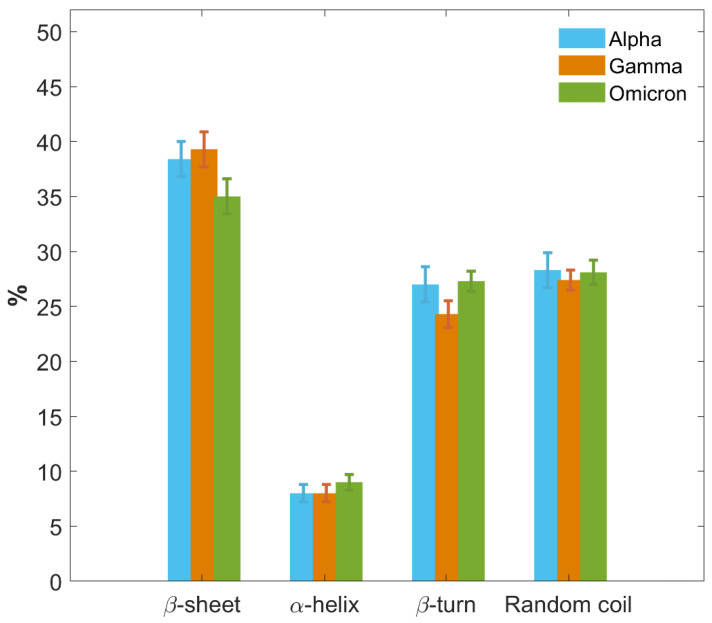
Histograms of secondary structure contents of S1 proteins for the three VoCs. Secondary structure percentage contents of S1 protein of the Alpha (blue), Gamma variant (orange) and Omicron variant (green) are graphically and comparatively reported. Results are calculated as the average of CD, IR and MD data. Uncertainties are calculated through the SD from average values.

In Figure 8, we further provide a graphical comparison of the secondary structure fractions for the S1 protein of each VoC. Results are reported as the weighted average of the outcomes obtained for each type of structure and each VoC from CD, IR and MD approaches. Moreover, a more detailed comparison of results provided separately by the three different techniques, along with their potentialities and limitations, is presented in SI, paragraph S7 and Figure S7).

The three VoCs show a similar secondary structure content, with some variations particularly concerning the Omicron S1 protein. All three proteins primarily consist of regions of disordered amino acid arrangements, with a random coil content around 28% for all of them, while the β-turn percentage is around 27% for Alpha and Omicron, and slightly lower in Gamma, around 24%.

Meanwhile, all VoCs show a low content of the α-helix structure, the same for all three S1 proteins within the error, between 8% and 9%.

A noticeable difference can be observed in the β-sheet content. Alpha and Gamma S1 proteins show the same percentage content of β-sheet within the error bars, specifically between 38% and 39%. The Omicron S1 protein instead shows a lower percentage of β-sheet content, approximately 35%, which represents a significant difference with respect to Alpha and Gamma beyond the error bars.

Moreover, Amide I band maxima (see Figure 3) are located at 1650, 1648 and 1647 cm^−1^ for the S1 proteins of Alpha, Gamma and Omicron variants, respectively. Note that the slight redshift of Amide I from Alpha to Omicron seems to follow the increasing number of mutations, which is smaller for Alpha (seven) and at the maximum for Omicron (thirty-one) (see Figure 1).

Focusing more deeply on the deconvoluted IR spectral components (see Figure 4 and Table S1), the Omicron Amide I clearly also shows a general slight shift to lower frequencies of all its components with respect to the other two protein variants.

The shift to lower frequencies in the Amide I vibration can typically be linked to stronger hydrogen bonding interactions with surrounding water molecules. This increased hydrogen bonding results in a decrease in the strength of the C=O vibration, leading to a redshift in the observed frequency [51,52,61] (see SI, Figure S8).

Our results would then suggest a more hydrophilic behavior of Omicron S1 protein [74,75]. From NPP ratio patches of proteins’ surfaces, Omicron RBD shows larger regions with lower NPP values, therefore a larger hydrophilic area, compared with Alpha RBD, which instead shows mostly neutral or hydrophobic regions (see Figure 6). Still, Omicron NTD shows wider hydrophilic areas compared with both Alpha and Gamma NTDs (see Figure 7).

Gravy values are found to be very similar between the three variants’ S1 proteins (Alpha Gravy value = −0.268, Gamma Gravy value = −0.246 and Omicron Gravy value = −0.268), as expected from their high similarity values. Indeed, as determined using the Pairwise Sequence Alignment Emboss Needle (https://www.ebi.ac.uk/Tools/psa/emboss_needle/, accessed on 27 March 2024), Alpha and Gamma present a similarity of 98.4%, Alpha and Omicron presented a similarity of about 96.8%, and finally Gamma and Omicron had a similarity of 95.6%. Protein alignments are reported in the SI (see pdf files in the SI and a graphical representation is provided in SI, paragraph S9, Figures S9–S11). Nevertheless, Gravy values depend only on amino acid sequences; therefore, the more hydrophilic behavior of the Omicron S1 protein revealed from NPP surface calculation and IR measurement suggests a difference in Omicron S1 protein 3D conformational structure, with respect to Alpha and Gamma S1.

The more hydrophilic character of the Omicron S1 protein may also explain its lower β-sheet content compared to Alpha and Gamma variants (see Figure 8). Indeed, the formation of β-sheet structures is known to be favored by hydrophobic amino acids and is usually associated with a higher average hydrophobicity value [74,75]. Moreover, due to the generally hydrophobic nature of protein structures, β-sheets tend to form in the buried region of the protein [76]. Therefore, the reduced presence of β-sheets in Omicron S1 could also be related to its tendency to assume an “open” conformation, more exposed to the surrounding solvent, as discovered through MD simulations. Computational results, indeed, revealed significant variations in the three VoCs’ R_g_ values (see Table 3). Alpha and Gamma S1 proteins, although initially modeled in an “open” configuration, tend to gradually fold and close on their structure, reducing their R_g_ values until reaching a more “closed” state with a final R_g_ value of (3.5 ± 0.3) nm and (3.06 ± 0.04) nm, respectively. Omicron, by contrast, is the only VoC that does not decrease its R_g_ value. Instead, it retains an “open” configuration when starting from an “open” model, with a final R_g_ value of (4.3 ± 0.1) nm [77].

This MD outcome suggests that the Omicron S1 protein appears to be more inclined to preserve its “open” configuration, in which the RBD and NTD domains are further apart than in the “closed” configuration (see Figure S2).

Assuming that the Omicron S1 protein can, on average, assume both “open” and “closed” states in water, while Alpha and Gamma S1 proteins predominantly assume a “closed” state, this conformational difference, due to the increasing number of mutations, may correlate with the lower content of β-sheet secondary structures and with the spectral differences observed in IR and CD spectra.

The “open” and “closed” configurations can be confidently associated with the “up” and “down” configurations of the RBD in the entire S protein, respectively [78,79,80,81]. Therefore, our results could suggest that the Omicron S protein exhibits greater stability in the RBD-up configuration compared to the Alpha and Gamma variants. Considering the infectious process, it is well established in the literature that the S protein assumes the RBDs in the “up” conformation to engage the ACE2 receptor on host cells, a prerequisite for viral entry and membrane fusion [82,83,84]. Our combined experimental and computational findings are consistent with a greater propensity of Omicron to bind the ACE2 receptor, which aligns with its higher level of affinity and infectivity with respect to other variants [18,21,24,32]. Actually, as shown in the literature [85], mutations in the Omicron S protein, and, in particular, its RBD, seem to generally facilitate a more efficient RBD ‘‘down’’ to ‘‘up’’ conformation and enhance ACE2 biding, in agreement with our results and observations. More specifically, Omicron S1 appears to favor a one-RBD-up conformation [18,86], which nevertheless yields stronger ACE2 interactions and supports prolonged viral attachment [18]. Additionally, the more hydrophilic behavior of the Omicron S1 protein, as revealed by IR spectroscopy and NPP surface ratio analysis, likely contributes to its enhanced receptor-binding capability. Some studies have highlighted the importance of hydration forces in complex stability, dynamic and binding kinetics, making the binding site accessible to both the ligand and receptor [87]. Still, in accordance with hydrophilic behavior, the mutations in the Omicron variant have also been recognized to cause a remarkable change in its S protein electrostatic potential surface, resulting in a more positive surface compared to previous VoCs (21). This represents an important evolutionary adaptation of the virus, enhancing the electrostatic interaction with the negatively charged surface of the ACE2 receptor and potentially increasing infectivity.

It is clear that the balance between hydrophilicity and hydrophobicity in the binding regions plays a primary role in the recognition and interaction between two proteins. Therefore, a quantification of both the hydrophilic and hydrophobic contribution, and therefore of the entire hydropathy profile, is useful to better characterize the interaction between two proteins. Moreover, it is known that the hydrophobicity/hydrophilicity distribution of viral proteins plays a role in the viral transmissibility [88,89,90], affecting the exposed surface area and the protein stability, especially if dealing with viruses which are transmitted through air (therefore, in a humid environment) [91,92].

## 4. Materials and Methods

### 4.1. Protein Preparation

Monomeric S1 subunits of S glycoprotein from three different variants of SARS-CoV-2 virus have been considered: Alpha variant S1, lineage B.1.1.7 (Cat. No. 40591-V08H12, aa 678, purity > 90%), Gamma variant S1, lineage P.1/P.1.1/P.1.2 (Cat. No. 40591-V08H14, aa 681, purity > 90%) and Omicron variant S1, lineage B.1.1.529 (Cat. No. 40591-V08H41, aa 678, purity > 95%). These have been provided by Sino Biological Europe GmbH (Eschborn, Germany). They were produced in baculovirus-infected insect cells, exhibiting a purity greater than 90%, as assessed by SDS-PAGE, and were subsequently used without additional purification. They differ from the S1 protein of SARS-CoV-2 wild type (WT) (Cat. No. 40591-V08B1, aa 681, purity > 90%), referred to as the variant that affected Wuhan in 2019, for a number of 7, 10 and 31 of mutations, respectively. Their amino acid sequences are reported in the SI (paragraph S1), and the mutations characterizing them are schematized in Table 5. Common mutations are reported with the same colors.

Each lyophilized protein was reconstructed by dissolving 100 µg of the pellet in distilled water (400 µL, pH 7.4) obtaining solutions with a concentration of 0.25 mg/mL. Other concentrations were investigated in our previous works [58,59], obtaining similar data and verifying the independence of IR measurements from concentrations.

### 4.2. Attenuated Total Reflection Infrared Spectroscopy and Data Analysis

IR spectra of the monomeric S1 proteins of the three SARS-CoV-2 VoCs were collected in ATR modality using a Bruker (Billerica, MA, USA) Vertex 70v Michelson spectrometer integrated with an ATR–Diamond single reflection module and a DLaTGS wide-range detector. Measurements were performed at room temperature (25 °C) and under vacuum conditions to minimize the spectral interferences due to IR absorption of water vapor and CO_2_. The background spectrum (water solvent) was collected shortly before each protein sample measurement, employing the same parameters and settings as those for the protein solution. For each ATR-IR measurement, 5 µL of the solution (protein solution or a water one) were directly dropped on the ATR diamond crystal, collecting seven spectral repetitions, each one made up of 128 scans, between 400 and 4000 cm^−1^, employing a spectral resolution of 2 cm^−1^. Following this strategy, five independent depositions were measured and analyzed for each protein sample. The ATR crystal was cleaned with ethanol (purity > 90%), distilled water and subsequently with a lens tissue in order to eliminate any spurious signal. OPUS 8.2. software (Bruker Optics, Billerica, MA, USA) and algorithms based on MATLAB (ver. 2018, MathWorks Inc., Natick, MA, USA), properly designed, were adopted to perform spectral analysis operations (absorbance calculation, baseline correction, water subtraction, ATR advanced correction, cut and average), as described in our previous works [58,59]. Protein secondary structures were investigated, focusing on the analysis of the Amide I vibrational absorption band [37,38,39], lying in the spectral range from 1590 to 1720 cm^−1^. For each protein, the Amide I band was normalized to the maximum value and deconvoluted into its spectral components. The calculation of 2nd-derivative absorption spectra allows us to achieve the frequencies of the convoluted spectral component, which were used as starting points for multiple gaussian fittings performed with OPUS 8.2 software, considering the residual error (RMSE) value as the goodness of fit parameter [55,56,58,59]. The estimation of proteins’ secondary structure percentage content is then achieved from the calculation of the percentage ratio of the area of each spectral component with respect to the integrated intensity of the total Amide I band (after the subtraction of side chain contribution [86]) [62,63,64,86].

The error associated with each secondary structure percentage content is calculated by propagating the standard deviations (SDs) of the percentage contribution of each spectral component, which is in turn obtained by adapting the final fit of the Amide I band to the spectra of each individual measurement run [62]. Further details about the IR analysis procedure are reported in the SI, paragraph S10, Figures S12 and S13.

### 4.3. Circular Dichroism Spectroscopy and Data Analysis

To analyze the chirality of monomeric S1 proteins through their optical activity, CD spectra were collected with an J715 Spectropolarimeter by Jasco (Tokyo, Japan), equipped with a high-intensity Xenon lamp, emitting in the UV region from 180 nm to 700 nm. An oscillating linear crystal creates circularly polarized light, and a photomultiplier tube converts the light signal in a current. A constant flow of nitrogen gas is used inside the spectropolarimeter in order to prevent the interferences of water vapor components. Samples are placed in a quartz suprasil cuvette (Hellma, Germany) with the optical path of 0.01 nm and it is thermalized at 25 °C with a Refrigerated Circulation Bath CBN 8-30 (Heto S.r.l. Brescia, Italy). Ethanol (CAS 64-17-5, Carlo Erba, purity > 90%) and distilled water are used to clean the cuvette. For each sample, the CD spectrum is the average of three independent CD measurements (meaning the cuvette has been filled with protein solution and washed three times), collected in step scanning mode, from 190 nm to 240 nm, with a 0.5 nm data pitch, a bandwidth of 1 nm, and the exposure time of 8 s. Raw data (measured in mdeg) are converted to the differential absorption coefficient (Δε) normalized on the amino acid number (according to the equation reported in S2). The instrument interfaces through the Jasco Spectra Manager^TM^ software (JASCO Corp., J-715, Rev. 1.00) which has been used for the first spectra processing (background subtraction, average and unit conversion). For the deconvolution, CD spectra fits are performed with the CDpro software package (https://sites.google.com/view/sreerama) [73,93]. This approach has been selected since CDPro is commonly employed for membrane protein analysis and for the estimation of secondary structure content [73]. Datasets were selected according to the type of proteins of interest (globular and membrane), and to the frequency range where spectra have been collected. It contains four algorithms for CD spectra analysis, namely CONTINLL, CDSSTR, SELCON3 and CLUSTR algorithms, and it can refer to 10 different datasets. For the deconvolution of S1 proteins’ CD spectra, CONTINLL, CDSSTR and SELCON3 algorithms were employed, with different reference basis sets, generally used for membrane and soluble proteins, namely SP37, SP43, SDP42, SDP48, SMP50 and SMP56. For each S1 protein CD spectrum, final fit is calculated as the average of fitting spectra obtained from each of the algorithms employing each of the basis sets. Also, secondary structure percentage contents were estimated as the average and SD of percentage content obtained from each of the algorithms employing each of the basis sets [73,93]. Further details about the CD analysis procedure are reported in the SI, paragraph S10, Figure S14.

### 4.4. ColabFold, Molecular Dynamics Simulation and Protein-Sol Software

Starting from the known FASTA amino acid sequences of S1 proteins, which are given by the provider, their three-dimensional (3D) structure is predicted, employing the AlphaFold2 [94] algorithm with the Many-against-Many sequence searching (MMseqs2) server [94], outperformed by ColabFOLD (v1.5.5: AlphaFold2 using MMseqs2, URL accessed on 21 November 2022). Structure files in pdb format were then visualized with PyMOL (ver. 3.0.5). These models of the S glycoprotein did not include glycosylation, which is expected to have minor effects on conformational dynamics.

These 3D models constitute the starting point for each variant for leading molecular dynamics (MD) simulations and post-processing analyses through the GROMACS v. 2022.3 package [64,95]. To perform MD simulations, the procedure is as follows: The COM of each protein is placed at the center of a cubic box of dimensions such that nearby images lay 10 Å away. The box is filled with TIP3P water molecules and 0.15 M of NaCl to make the whole system neutral. CHARMM and AMBER are among the most widely used force fields for protein simulations and, in particular, the CHARMM force field has been employed in several studies concerning the S protein [96,97,98]. More specifically, for all three S1 proteins, we opted for CHARMM22/CMAP, owing to its successful application in earlier studies dealing with vibrational and IR protein features. Furthermore, thanks to the addition of the CMAP term [65,95,96,97,98,99] in the dihedral potential, the CHARMM22/CMAP force field is able to reproduce the transitions between α-helix and β-sheet secondary structures and to keep a reasonable balance among the α-helix and random coil. Additionally, the CHARMM22* force field incorporates the Urey–Bradley term [91,99,100,101], which accounts for angle bending through 1,3 nonbonded interactions. This feature enhances the accuracy of molecular vibration assessments with respect to other commonly used force fields.

Each simulation is run as follows: A minimization phase was performed, for 5 × 10^3^ + 5 × 10^3^ steps of steepest descent and conjugate gradient algorithms in series, with a maximum force value of 10 kJ·mol^−1^·nm^−1^. The LINCS algorithm [102] is employed to impose constraints on all the hydrogen bonds in order to reduce the degrees of freedom. Then, the equilibration strategy consists of (i) 3 × 20 ns in the NVT ensemble at different temperatures (150, 200 and 300 K), with position restraints on the protein backbone and side chains to relax the solvent around the protein, and (ii) 20 ns in the NpT ensemble without restraints. Still, once equilibrated, for a single replica of each model, simulations were performed in the NpT ensemble for 600 ns. Although this simulation time scale may not capture slower, large-scale conformational changes, this limitation does not affect the comparison of secondary structure between variants, as the MD results are in good agreement with experimental data (see Results and Discussion Sections).

The temperature of the whole system was kept fixed at room temperature using the V-rescale thermostat [103], with a coupling time of 0.1 ps, and the pressure was kept fixed at 1 bar using the Parrinello–Rahman barostat [104,105] with a coupling time of 2 ps and an isotropic compressibility of 4.5 × 10^−5^ bar^−1^. The Particle Mesh Ewald (PME) algorithm [106] is employed to menage the Coulomb interaction. A time step of 2 fs and a non-bonded pair list cut-off of 1.0 nm were used. The list was updated every 10 steps. Final data obtained from MD simulations were analyzed using GROMACS and Python v. 3.12 [107] handmade programs and the Visual Molecular Dynamics v. 1.9.3 tool [108], considering only the last 100 ns of the simulations, when the systems had reached equilibrium. In particular, concerning FES heatmaps, these have been computed for the RMSD and the R_g_ and the color scale corresponds to the free energy, representing the probability that the model assumes that specific position in the phase space [Rg; RMSD], meaning the probability to assume those specific values of the two quantities. The free energy is defined in correlation with the canonical partition function (in the logarithmic term) in the following way:

$$G=-0.001\cdot Av\cdot Kb\cdot T\cdot (\log_{10}\left(Z\right)-\log_{10}\left(max(Z))\right)$$

G is expressed in kcal/mol, with Kb = 3.2976268 × 10^−24^ (cal/K) being the Boltzmann constant, Av = 6.0221417923 is Avogadro’s number, and T = 298 (K) is the temperature. Square matrix Z contains the frequency of occurrence of the pairs of RMSD and R_g_ values.

Protein-sol software (https://protein-sol.manchester.ac.uk/, accessed on 14 March 2025) was employed to compute the non-polar to polar (NPP) ratio surface [69] of S1 proteins in order to obtain information and the visualization of the protein hydrophobicity profile [68]. Results are reported as colormaps (see Figure 6 and Figure 7) in a scale range going from NPP = 2.5 (green, non-polar) to NPP = 0.4 (purple, polar).

## 5. Conclusions

In this work, we present, to the best of our knowledge, the first systematic and comparative structural investigation of the monomeric S1 proteins for the Alpha, Gamma, and Omicron variants of the SARS-CoV-2 virus. Our analysis focuses on the S1 domains of these VoCs, which play a major role in mediating viral attachment to the host ACE2 receptor. Carried out at physiological pH (7.4), this study reveals noticeable structural alterations arising from amino acid mutations, in terms of secondary structure composition, hydrophobicity, and the overall conformational organization of the S1 domain.

By combining the results from IR Amide I band absorption, CD signal and MD simulations, the secondary structure content has been determined for the S1 proteins of the three VoCs, in terms of *β*-sheet, random coil, *α*-helix and *β*-turn contents, demonstrating that S1 proteins of Alpha, Gamma, and Omicron variants share highly similar secondary structure contents, as expected from their high similarity in amino acid sequences and the limited number of mutations. Nevertheless, a significant difference beyond experimental error was detected in the slight reduced β-sheet content of Omicron S1 protein compared to Alpha and Gamma. Moreover, the redshift, observed in Omicron Amide I band components, with respect to those of Alpha and Gamma, can be associated with its higher hydrophilic character, which is further testified by the NPP ratio surface computed for RBD and NTD domains of the three S1 proteins, i.e., the regions where most mutations occur. In particular, the Omicron S1 protein presents an overall more hydrophilic character, showing larger high-NPP-ratio areas, and this is also consistent with the reduced β-sheet content of Omicron S1. IR and CD spectral differences further suggest a variation in the 3D conformation of the Omicron S1 protein, coherently supported by computational results showing that Omicron S1 is the only variant capable of strongly retaining the “open” conformation, whereas Alpha and Gamma progressively collapse into a “closed” configuration in nearly 600 ns.

This greater tendency of Omicron S1 to keep an “open” conformation can be confidently related to the configuration “RBD-up” that S protein assumes when it anchors the ACE2 receptor, therefore suggesting and explaining the stronger affinity of Omicron S1 for ACE2, compared to Alpha and Gamma.

In conclusion, our results combine different techniques, both experimental and computational, for the understanding of the conformational structure of protein domains with high amino acid similarity, and for the research of structural changes induced by very few mutated amino acids. The knowledge of the structural characteristics of SARS-CoV-2 S1 variant proteins represents a crucial step in the development of effective therapeutic protocols and/or prophylaxis and monitoring strategies and is of primary importance for understanding and addressing further surveillance, preventive and monitoring actions.

## Figures and Tables

**Table 1:** Secondary structure percentage content from CD analysis. Percentage fractions of the secondary structure of monomeric S1 protein of SARS-CoV-2 Alpha, Gamma and Omicron variants are estimated from CD spectra deconvolution performed with the CDSSTR, SELCON3 and CLUSTR algorithms using SP37, SP43, SDP42, SDP48, SMP50 and SMP56 basis sets.

Variants	β-Sheets (%)	α-Helix (%)	β-Turn (%)	Random Coil (%)
Alpha	38 ± 7	8 ± 4	23 ± 7	31 ± 7
Gamma	39 ± 6	11 ± 6	20 ± 2	30 ± 4
Omicron	37 ± 7	8 ± 4	23 ± 3	32 ± 5

**Table 2:** Secondary structure percentage content from IR analysis. Percentage values were estimated from Amide I band deconvolution with a multi-Gaussian fit for monomeric S1 protein of SARS-CoV-2 Alpha, Gamma, and Omicron variants. Percentage data are presented as MEAN ± SD.

Variants	β-Sheets (%)	α-Helix (%)	β-Turn (%)	Random Coil (%)
Alpha	39 ± 2	8 ± 1	25 ± 3	28 ± 2
Gamma	40 ± 3	9 ± 1	24 ± 2	27 ± 1
Omicron	35 ± 3	11 ± 1	27 ± 1	27 ± 1

**Table 3:** Average Radius of Gyration (Rg) values in nm for the three variants of S1 proteins. Results are reported for Alpha, Gamma and Omicron both in closed and open states. Values are obtained as the MEAN ± SD over the last 100 ns of MD simulations.

Variants	Radius of Gyration (nm)			
	Closed State		Open State	
	Initial	Final	Initial	Final
Alpha	3.6 ± 0.1	3.33 ± 0.04	4.2 ± 0.3	3.5 ± 0.3
Gamma	3.20 ± 0.07	3.12 ± 0.02	3.5 ± 0.2	3.06 ± 0.04
Omicron	3.31 ± 0.05	3.18 ± 0.03	4.2 ± 0.2	4.3 ± 0.1

**Table 4:** Secondary structure percentage content from MD analysis. Results for Alpha, Gamma and Omicron variants in the “closed” conformation and the “open” conformation in parenthesis are reported. Percentage values are calculated as the MEAN ± SD over the last 100 ns of simulation.

Variants	β-Sheets (%)	α-Helix (%)	β-Turn (%)	Random Coil (%)
Alpha	35 ± 3 (31 ± 3)	6 ± 1 (5 ± 1)	30 ± 2 (27 ± 2)	28 ± 3 (35 ± 3)
Gamma	38 ± 1 (39 ± 1)	7 ± 1 (5 ± 1)	27 ± 2 (27 ± 2)	27 ± 2 (28 ± 2)
Omicron	35 ± 2 (36 ± 2)	8 ± 1 (5 ± 1)	27 ± 2 (29 ± 2)	29 ± 2 (28 ± 2)

**Table 5:** Variants of Concern (VoCs) and their mutations. Their standard nomenclature, lineages and mutations with respect to Wuhan wild type (WT) virus S1 protein (Cat. No. 40591-V08B1) are listed. Legend: A(xyz)B means that amino acid A in position (xyz) has been substituted with amino acid B. A(xyz)del means that amino acid A in position (xyz) has been deleted. ins(xyz)ABC means that the insertion of ABC amino acids in position (xyz) has occurred. Colors and/or underline are used to indicate mutations in common among the proteins.

Variants	Lineage	Mutations
Alpha	B.1.1.7	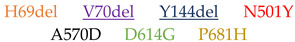
Gamma	P.1/P.1.1/P.1.2	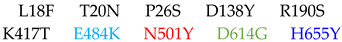
Omicron	B.1.1.529	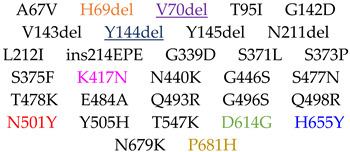

## Data Availability

The data presented in this study are available on request from the corresponding authors.

## Supplementary Materials

The following supporting information can be downloaded at https://www.mdpi.com/article/10.3390/ijms262110342/s1.
