# Supplementary material for: Multimodal Structural Characterization of SARS-CoV-2 Spike Variants: Spectroscopic and Computational Insights
Source: Int J Mol Sci. 2025 Oct 23;26(21):10342. doi: 10.3390/ijms262110342 (PMC12609920; doi:10.3390/ijms262110342)
Supplement: Supplementary file 1 [file ijms-26-10342-s001.zip › ijms-3893381-supplementary.pdf]

Supporting Information

Title: Multimodal Structural Characterization of SARS-CoV-2 Spike Variants: Spectroscopic and Computational Insights

Tiziana Mancini[a,], Nicole Luchetti[b], Salvatore Macis[a], Velia Minicozzi[c], Rosanna Mosetti[d], Alessandro Nucara[a,*], Stefano Lupi[a] and Annalisa D'Arco[a]*

[a] Department of Physics, University La Sapienza, P.le A. Moro 2, 00185, Rome, Italy

[b] Engineering Department, Università Campus Bio-Medico di Roma, Via Alvaro del Portillo 21, 00128, Rome, Italy

[c] Department of Physics, University of Rome Tor Vergata, Via della Ricerca Scientifica 1, 00133 Rome, Italy

[d] Department of Basic and Applied Sciences for Engineering (SBAI). Sapienza University of Rome, Via A. Scarpa 16, 00161, Rome, Italy

Correspondence

Tiziana Mancini, Annalisa D'Arco

Department of Physics, University La Sapienza, P.le A. Moro 2, 00185, Rome, Italy

E-mail: tiziana.mancini@uniroma1.it, alessandro.nucara@uniroma1.it

S1. Amino acid sequences of SARS-CoV-2 variants S1 proteins

Alpha variant S1 protein sequence

VNLTTRTQLPPAYTNSFTRGVYYPDKVFRSSVLHSTQDLFLPFFSNVTWFHAISGTNGTKRFDNPVLPF
NDGVYFASTEKSNIIRGWIFGTTLDSKTQSLLIVNNATNVVIKVCEFQFCNDPFLGVYHKNNKSWMES
EFRVYSSANNCTFEYVSQPFLMDLEGKQGNFKNLREFVFKNIDGYFKIYSKHTPINLVRDLPQGFSALE
PLVDLPIGINITRFQTLLALHRSYLTPGDSSSGWTAGAAAYYVGYLQPRTFLLKYNENGTITDAVDCALD
PLSETKCTLKSFTVEKGIYQTSNFRVQPTESIVRFPNITNLCPFGEVFNATRFASVYAWNRKRISNCVAD
YSVLYNSASFSTFKCYGVSPTKLNDLCFTNVYADSFVIRGDEVRQIAPGQTGKIADYNYKLPDDFTGCV
IAWNSNNLDSKVGGNYNYLYRLFRKSNLKPFERDISTEIYQAGSTPCNGVEGFNCYFPLQSYGFQPTY
GVGYQPYRVVVLSFELLHAPATVCGPKKSTNLVKNKCVNFNFNGLTGTGVLTESNKKFLPFQQFGRDI
DDTTDAVRDPQTLEILDITPCSFGGVSVITPGTNTSNQVAVLYQGVNCTEVPVAIHADQLTPTWRVYST
GSNVFQTRAGCLIGAEHVNNSYECDIPIGAGICASYQTQTNSHRRARAHHHHHHHHHH

Gamma variant S1 protein sequence
VNFTNRTQLPSAYTNSFTRGVYYPDKVFRSSVLHSTQDLFLPFFSNVTWFHAIHVSGTNGTKRFDNPV
LPFNDGVYFASTEKSNIIRGWIFGTTLDSKTQSLLIVNNATNVVIKVCEFQFCNYPFLGVYYHKNNKSW
MESEFRVYSSANNCTFEYVSQPFLMDLEGKQGNFKNLSEFVFKNIDGYFKIYSKHTPINLVRDLPQGF
SALEPLVDLPIGINITRFQTLLALHRSYLTPGDSSSGWTAGAAAYYVGYLQPRTFLLKYNENGTITDAVD
CALDPLSETKCTLKSFTVEKGIYQTSNFRVQPTESIVRFPNITNLCPFGEVFNATRFASVYAWNRKRISN
CVADYSVLYNSASFSTFKCYGVSPTKLNDLCFTNVYADSFVIRGDEVRQIAPGQTGTIADYNYKLPDDF
TGCVIAWNSNNLDSKVGGNYNYLYRLFRKSNLKPFERDISTEIYQAGSTPCNGVKGFNCYFPLQSYG
FQPTYGVGYQPYRVVVLSFELLHAPATVCGPKKSTNLVKNKCVNFNFNGLTGTGVLTESNKKFLPFQ
QFGRDIADTTDAVRDPQTLEILDITPCSFGGVSVITPGTNTSNQVAVLYQGVNCTEVPVAIHADQLTPT
WRVYSTGSNVFQTRAGCLIGAEYVNNSYECDIPIGAGICASYQTQTNSPRRARAHHHHHHHHHH

Omicron variant S1 protein sequence
VNLTTRTQLPPAYTNSFTRGVYYPDKVFRSSVLHSTQDLFLPFFSNVTWFHVISGTNGTKRFDNPVLPF
NDGVYFASIEKSNIIRGWIFGTTLDSKTQSLLIVNNATNVVIKVCEFQFCNDPFLDHKNNKSWMESEF
RVYSSANNCTFEYVSQPFLMDLEGKQGNFKNLREFVFKNIDGYFKIYSKHTPIIVREPEDLPQGFSALE
PLVDLPIGINITRFQTLLALHRSYLTPGDSSSGWTAGAAAYYVGYLQPRTFLLKYNENGTITDAVDCALD
PLSETKCTLKSFTVEKGIYQTSNFRVQPTESIVRFPNITNLCPFDEVFNATRFASVYAWNRKRISNCVAD
YSVLYNLAPFFTFKCYGVSPTKLNDLCFTNVYADSFVIRGDEVRQIAPGQTGNIADYNYKLPDDFTGCV
IAWNSNKLDSKVSGNYNYLYRLFRKSNLKPFERDISTEIYQAGNKPCNGVAGFNCYFPLRSYSFRPTY
GVGHQPYRVVVLSFELLHAPATVCGPKKSTNLVKNKCVNFNFNGLKGTGVLTESNKKFLPFQQFGRD
IADTTDAVRDPQTLEILDITPCSFGGVSVITPGTNTSNQVAVLYQGVNCTEVPVAIHADQLTPTWRVYST
GSNVFQTRAGCLIGAEYVNNSYECDIPIGAGICASYQTQTKSHRRARAHHHHHHHHHH

S2. Conversion formula from Molar Ellipticity θ [mdeg] to normalized Differential Absorption Coefficient Δε

$$\Delta\varepsilon = (\theta / 3298) / AA$$

where AA is the number of amino acids

S3. CD spectral absorption between 190 and 240 nm of S1 proteins from WT SARS-CoV-2 virus and Alpha, Gamma and Omicron VoCs.

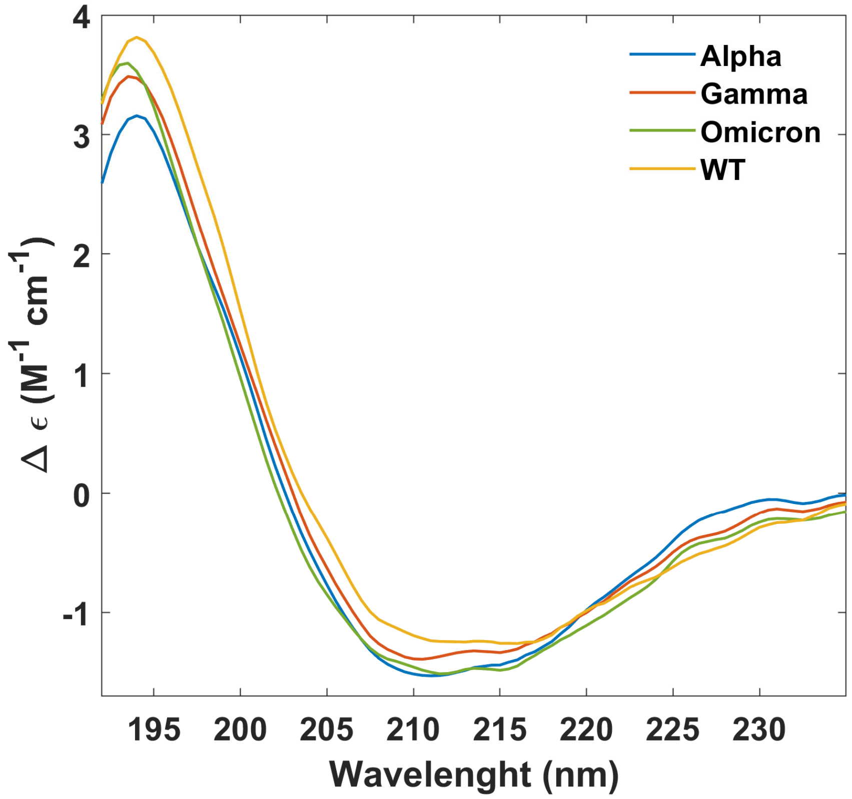

Figure S1. CD spectra between 190 and 240 nm of monomeric S1 protein of Alpha (blue), Gamma (orange) and Omicron (green) variants of SARS-CoV-2 virus compared with WT (yellow).

S4. Amide I bands deconvolution in to gaussian components of S1 proteins from the three VoCs and their assignment to secondary structure vibrations.

Alpha		Gamma		Omicron		Assignment
Frequency (cm^{-1})	Integrated intensity (%)	Frequency (cm^{-1})	Integrated intensity (%)	Frequency (cm^{-1})	Integrated intensity (%)	
		1607	0.7			Side chain
1609	2.1			1610	3.2	Side chain
1624	14.7	1624	19.4	1624	14.9	β-sheet
				1634	13.5	β-sheet
1638	21.1	1637	18.9			β-sheet
				1643	15.6	Random coil
		1645	8.5			Random coil
1647	13.2					Random coil
		1651	1.4	1651	10.3	Random coil
1654	14.5	1654	17.0			Random coil
				1658	10.3	α-helix
		1661	8.6			α-helix
1662	7.9					α-helix
				1665	13.0	β-turn
1671	12.9	1670	12.4			β-turn
				1673	4.6	β-turn
		1675	2.3			β-turn
				1679	8.5	β-turn
1683	11.0	1684	8.8			β-turn
				1690	4.6	β-sheet
				1696	1.1	β-sheet
1697	2.4	1698	1.8			β-sheet
				1704	0.2	β-sheet

Table S1. Amide I absorption peaks of S1 proteins from the three VoCs. Secondary structure assignment SARS-CoV-2 Variants of Concern Alpha, Gamma and Omicron for S1 units derived from the Gaussian decomposition of the vibrational absorption spectra.

S5. 3D visualization with PyMOL of "Closed" and "Open" state of Omicron S1 protein

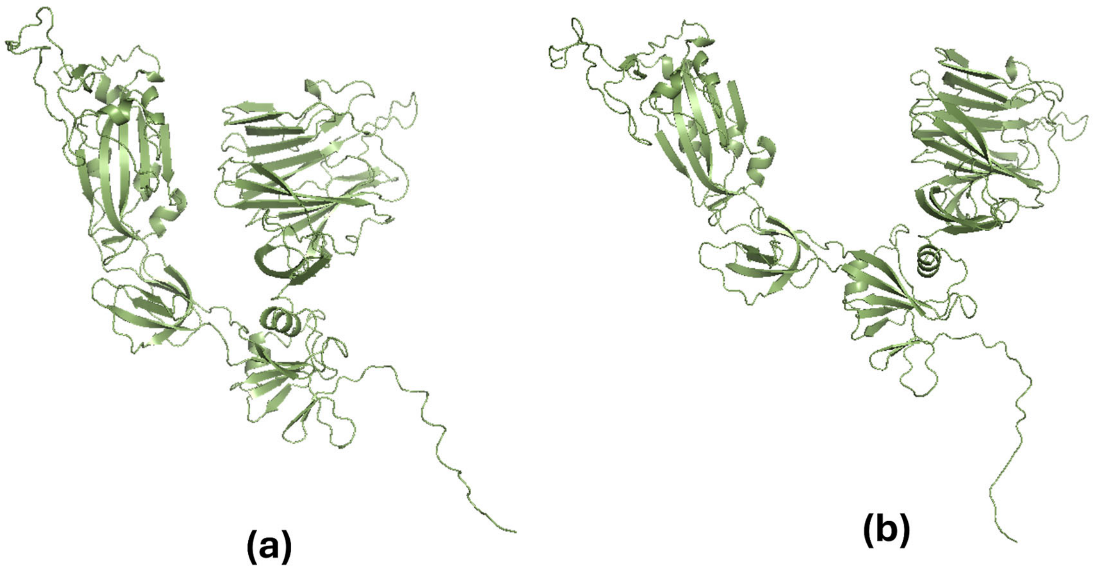

Figure S2. 3D Visualization of monomeric S1 protein from Omicron variant in its (a) "closed" and (b) "open" states. Models are obtained with AlphaFOLD2 and are visualized with PyMOL.

Variant and state	Starting distance (nm)	Average distance (nm)
Alpha closed	4.7	4.7 ± 0.1
Alpha opened	6.8	6 ± 1
Gamma closed	3.9	4.2 ± 0.1
Gamma opened	6.4	3.7 ± 0.2
Omicron closed	4.6	4.1 ± 0.1
Omicron opened	7.1	8.5 ± 0.3

Table S2. Distance between the centres of mass of the NTD and RBD domains for both "open" and "closed" states of S1 proteins of Alpha, Gamma and Omicron variants. Both the initial distance and the average distance (calculated over the last 100 ns of simulations) are reported.

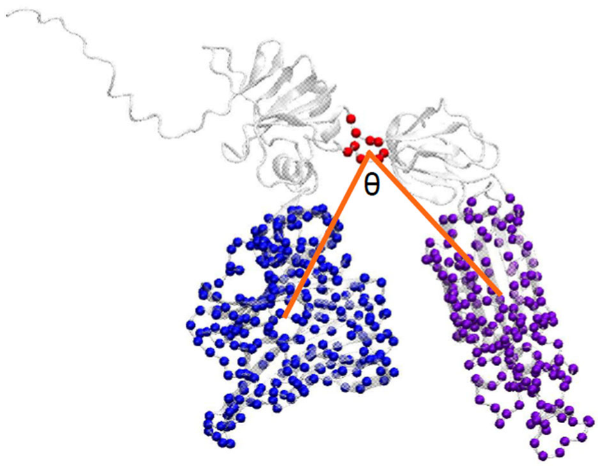

Figure S3. Angle θ between the vectors identified by the COM of the selected points (example of alpha closed model). Red points: Cα atoms of residues 571–574 and 301–305. Blue points: the Cα atoms of residues 1–270. Violet points: Cα atoms of residues 315–500.

Variant	Starting angle	Average angle
Alpha closed	63°	(66 ± 3)°
Alpha open	93°	(83 ± 21)°
Gamma closed	55°	(59 ± 2)°
Gamma open	92°	(51 ± 2)°
Omicron closed	62°	(60 ± 1)°
Omicron open	100°	(117 ± 7)°

Table S3. Values of angle θ for both “open” and “closed” states of S1 proteins of Alpha, Gamma and Omicron variants. Both the initial and the average angle (calculated over the last 100 ns of simulations) are reported.

S6. Root Mean Square Deviation (RMSD), Radius of Gyration (Rg) and Root Mean Square Fluctuations (RMSF) calculated from MD simulation for Alpha, Gamma and Omicron variants S1 proteins both for the initial “closed” and “open” states.

Model	RMSD (nm)	
	closed	open
Alpha	0.99±0.06	1.23±0.19
Gamma	1.18±0.02	1.55±0.03
Omicron	1.01±0.05	1.12±0.12

Table S4. RMDS values calculated as the average on the last 100 ns of MD simulation of S1 proteins of Alpha, Gamma and Omicron variant, both in initial “closed” and “open”states.

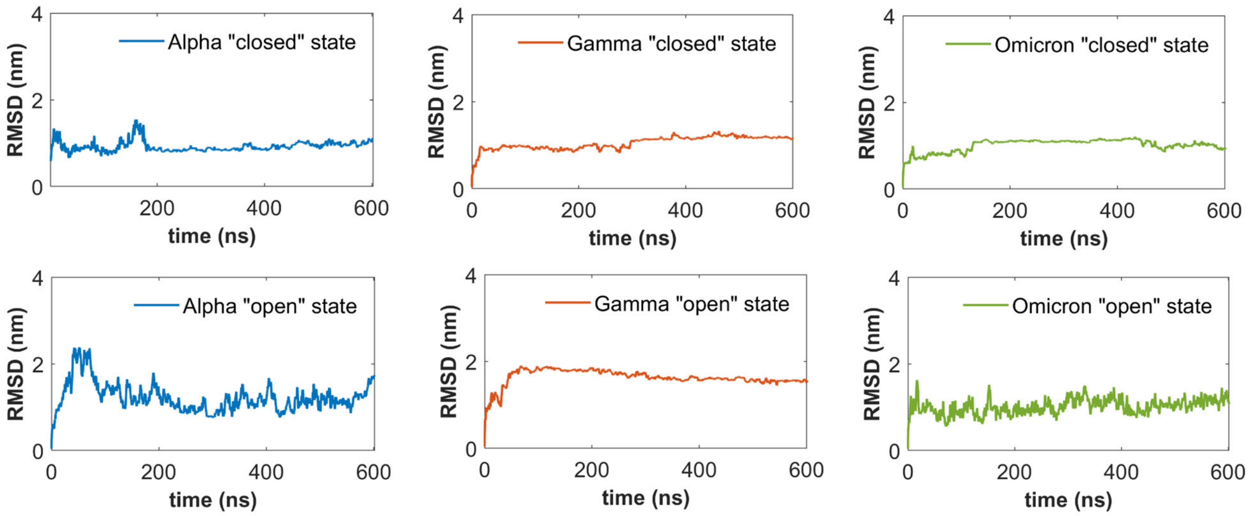

Figure S4. RMSD curves during 600 ns of MD simulations of S1 protein of Alpha (blue curves), Gamma (orange curves) and Omicron (green curves) variant, starting both from the “closed” (top panels) and the “open” (bottom panels) state for each variant

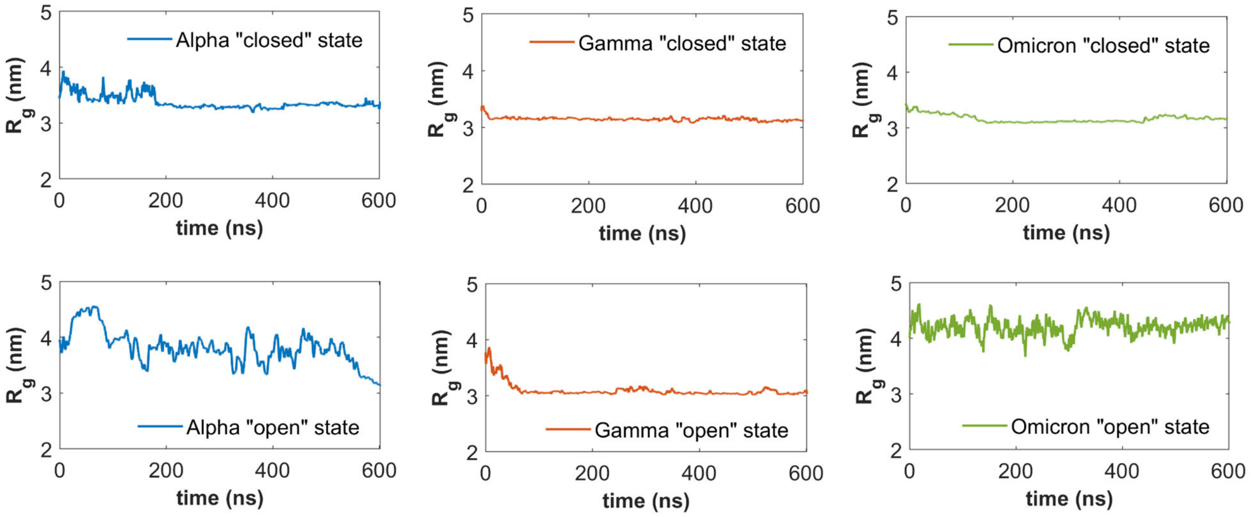

Figure S5. Rg curves during 600 ns of MD simulations of S1 protein of Alpha (blue curves), Gamma (orange curves) and Omicron (green curves) variant, starting both from the "closed" (top panels) and the "open" (bottom panels) state for each variant

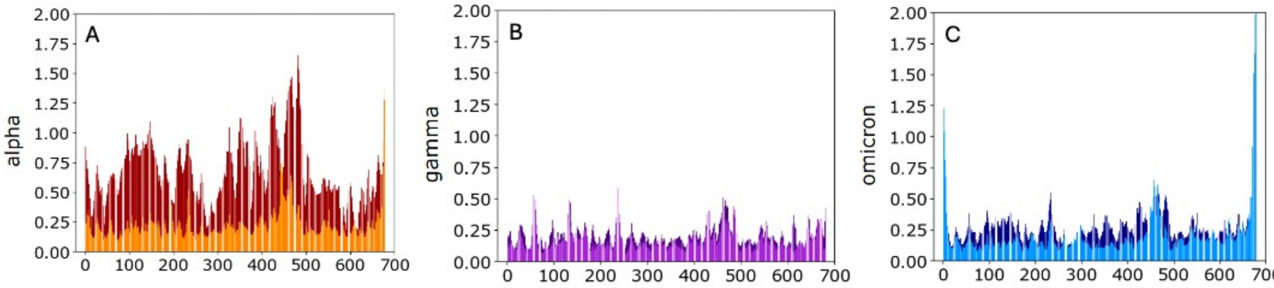

Figure S6. RMSF for Ca atoms of Alpha, Gamma and Omicron variants. Dark curves are for the "open" and light curves for the "closed" configurations.

S7. Secondary structure content of S1 proteins for Alpha, Gamma and Omicron variants. Comparison between estimation obtained with the three different techniques: IR spectroscopy, MD simulations and CD spectroscopy, their potentialities and limitations.

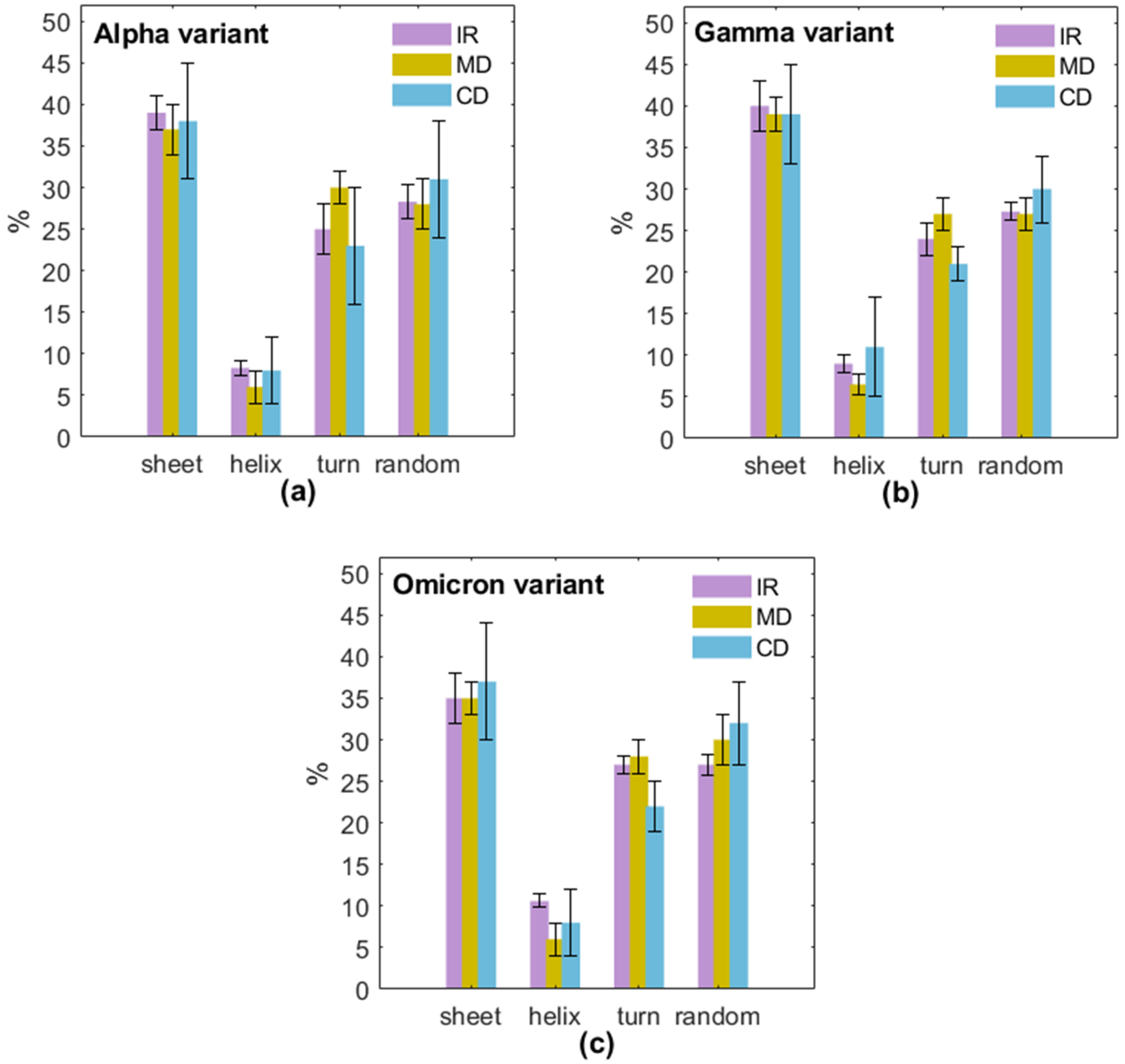

Figure S7. Histograms of secondary structure contents estimated with three different techniques. IR spectral data (violet), MD simulations (ochre) and by CD spectral data (cyan) are graphically reported for (a) Alpha variant, (b) Gamma variant and (c) Omicron variant S1 proteins. Error bars in the experimental and modelling data are estimated as explained in the Materials and Methods section.

Fig. S7 shows the comparison between the secondary structure fractions of S1 protein of Alpha, Gamma and Omicron variants and resulting from the three different approaches: IR spectral analysis, CD spectral analysis and MD simulations.

In particular, observing our IR spectroscopic results, for both Alpha, Gamma and Omicron proteins, the secondary structure is mostly constituted by disordered components, with a β-turn percentage content between 24 % and 27 % and a random coil content around 27 % and 28 %. Moreover, Alpha, Gamma and Omicron S1 proteins show a high content of β-sheet structure, between 35 % and 40 %, and low content of α-helix structure, between about 8 % and 11 %.
From CD spectroscopy analysis we observed similar secondary structure contents compared with IR analysis. Also from CD measurements, variants S1 proteins result to be mostly made up of a disordered structure, with a percentage content of β-turns between 20 % and 23 % and a random coil content between 30 % and 32 %. Moreover, the β-sheet percentage content is around 37 % and 39 %, and the α-helix content is between 8 % and 11 %, for all three S1 variants.
Finally, the employment of high accuracy computational methods for the prediction of 3D protein structures from the amino acids sequence, such as MD simulations, allowed us to compare the experimental data with theoretical ones. From MD simulations, β-sheet content is between 35 % and 39 % for all S1 three variants. α-helix is instead estimated to be around 6 % and 7 % for all three proteins, slightly less if compared with IR and CD estimations. Meanwhile, also from MD simulations, disordered structures constitute most of the S1 proteins structures, with a β-turn content between 27 % and 29 % and a random coil content between 27 % and 30 %, for all three variants S1 proteins.
Observing the cyan bars in Fig. S7, secondary structure content estimated from CD spectral data shows much larger error bars with respect to IR and MD analysis results, shown in purple and ochre, respectively. Indeed, as it is known from literature, CD spectroscopy provide a more accurate measure of α-helix content, due to the well-defined chiral signal due to the helical structure. Instead, β-turn structure generally has a less defined CD signal, because of its more disordered nature. Meanwhile, due to the limited instrument sensitivity and the solvent absorption below 190 nm (40-42), CD spectroscopy does not allow a highly sensitive estimation of β-sheet structure content, whose main absorption peaks are located in the low wavelength spectral region (40).
Generally, IR secondary structure estimation is more sensitive, in particular in the central spectral region of amide I vibrational band, where α-helix and random coil contributions are found. Because of spectral processing tools, such as cutting and baseline correction, the absorption peaks located at low frequencies and at high frequencies of the amide interval (1600-1710 cm^{-1}), can be affected by a larger uncertainty. It is where β-sheet and β-turn vibrations lie and from our IR analysis, β-sheet and β-turn content estimations actually have a slightly larger error if compared to α-helix and random coil one.
For what concerns MD simulations, while they provide detailed insights into protein dynamics and conformational variability, it has to be specified that they are limited by the timescales accessible and by the accuracy of the chosen force field. Subtle energetic effects or rare events might not be fully captured within the simulation window. Moreover, the accuracy of our conclusions depends on the quality of the starting structures and the extent of sampling.
To address these limitations and strengthen the findings, possible improvement could be apply, such as: enhancing sampling techniques (e.g., metadynamics, replica exchange), to better capture transitions between conformational states, and running longer or multiple MD simulations to improve statistical robustness.
Anyway, in conclusion, while we did not find such remarkable differences in secondary structure content, IR spectra together with MD simulations can provide more sensitive

interpretations compared to CD spectroscopy, on which other kinds of structural change are occurring for the three variants.

S8. Graphical scheme of Omicron "open" model and its IR spectral features

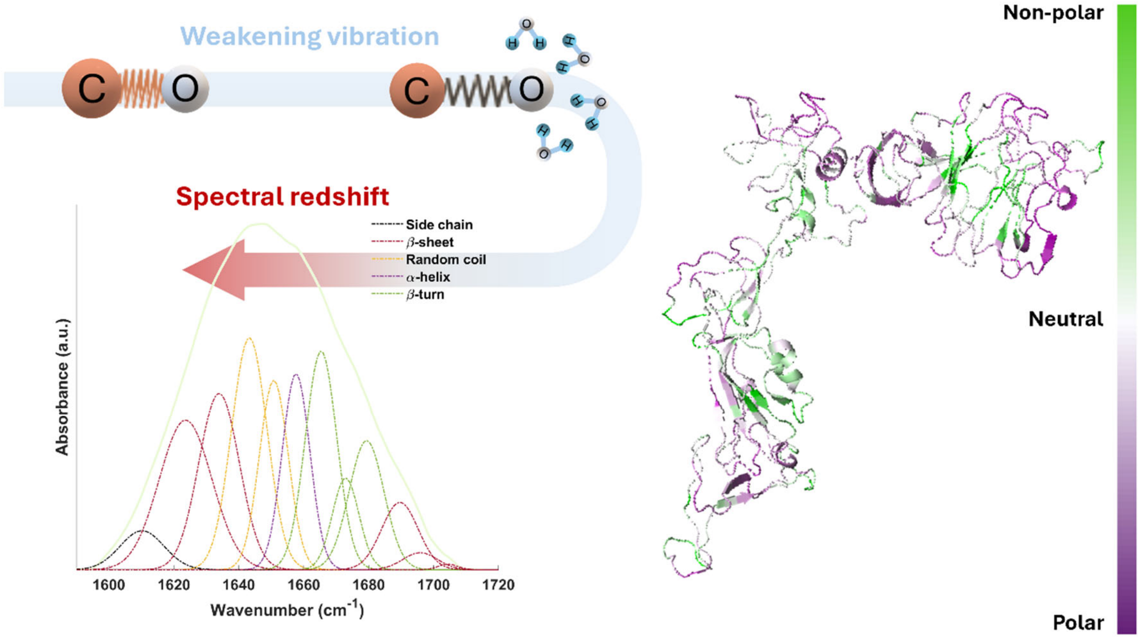

Figure S8. Graphical representation of the relation between the larger hydrophilicity of Omicron S1 protein "open" model and the redshift of its IR spectral component. The "open" configuration shows larger polar areas (purple ones); the bonding between amino acid C=O groups and water molecules induces to weakening of C=O vibrations and therefore to a redshift of its absorption bands.

S9. Spatial alignment of variants proteins final 3D models

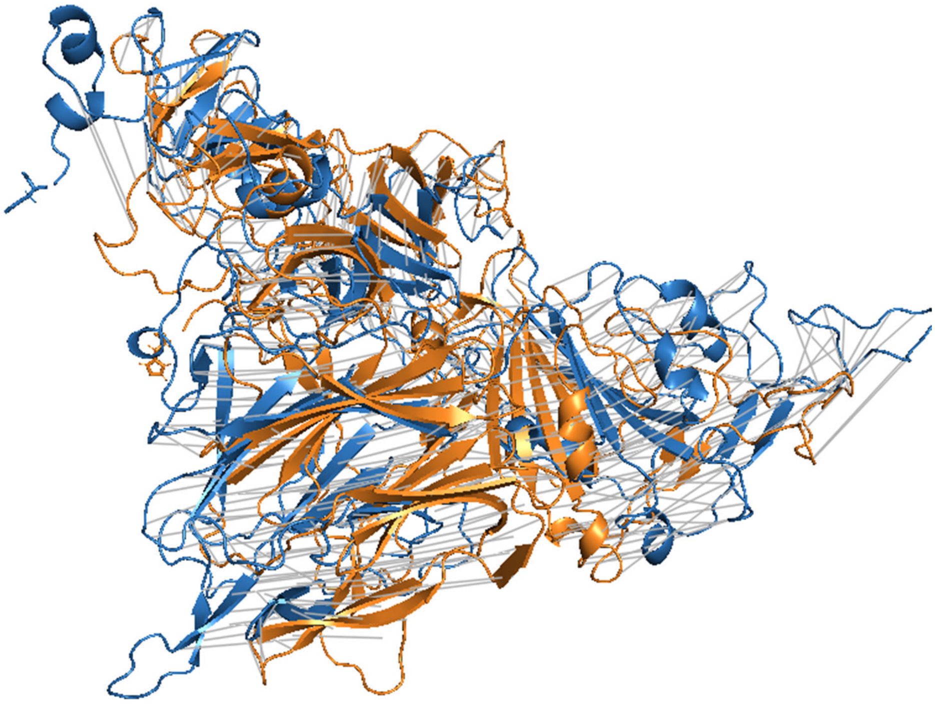

Figure S9. Spatial alignment Alpha (blue) and Gamma (orange) 3D models of S1 proteins at the end of 600 ns MD simulations.

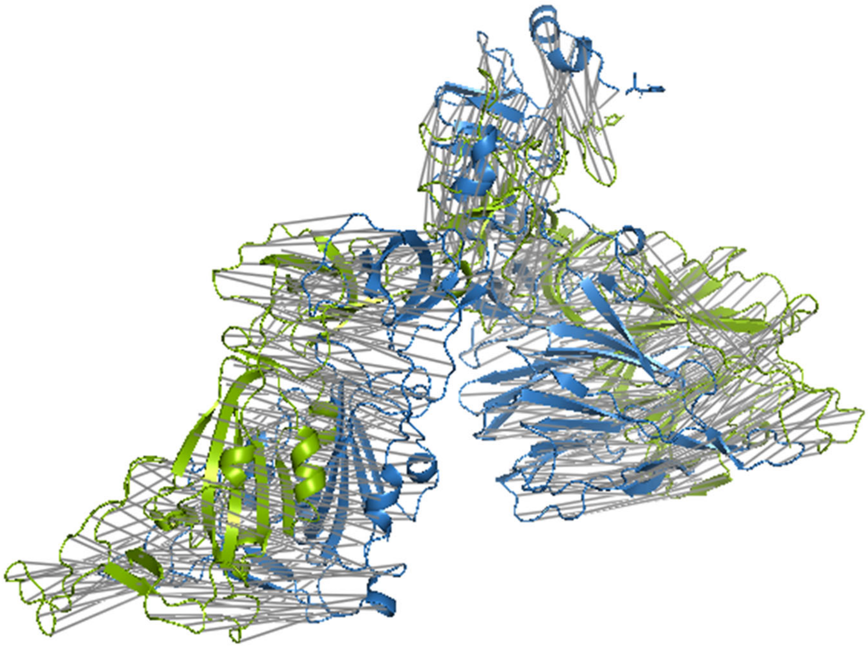

Figure S10. Spatial alignment Alpha (blue) and Omicron (green) 3D models of S1 proteins at the end of 600 ns MD simulations.

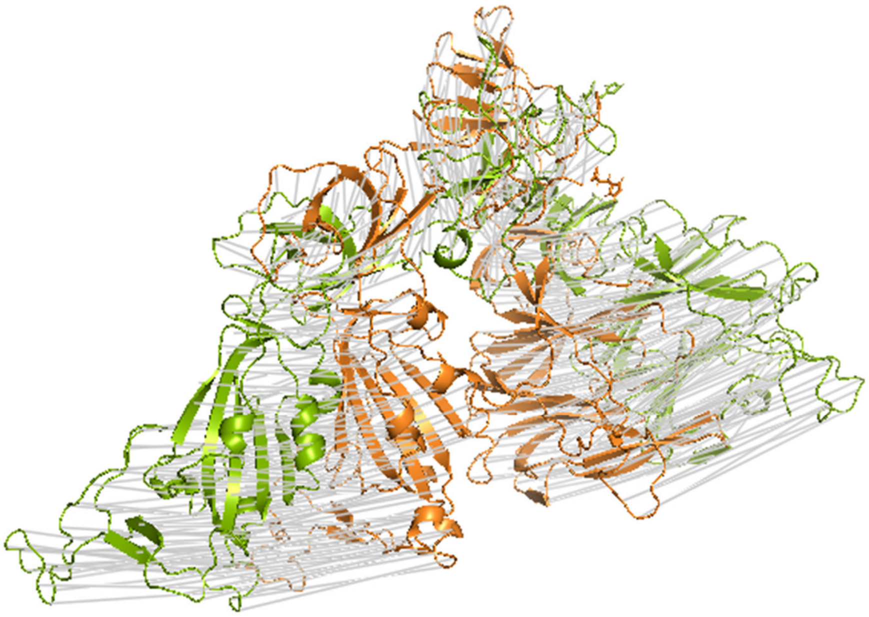

Figure S11. Spatial alignment Gamma (orange) and Omicron (green) 3D models of S1 proteins at the end of 600 ns MD simulations.

S10. Details on ATR-IR and CD spectra deconvolution methodology

The spectral analysis of ATR-IR spectra of proteins in H_2O solution particularly focused on the amide I vibrational absorption band, lying in the spectral range between 1590-1720 cm^{-1} and mainly attributed to the stretching vibration of C=O group present in each amino acid. The energy of this vibration heavily depends on the local protein structure, both because C=O vibration is affected by the surrounding H-bonding networks, which in turn are related to the local molecular 3D arrangement, and because the ordered structures that proteins assume allow the exciton couplings among C=O in different amino acids and the delocalization of the vibrational mode. This one therefore assumes a specific energy, distinctively attributable to each type of structure. From this, it results that the spectral analysis of amide I band can provide both the estimation of protein secondary structure content and deep information about conformational order and hydrophilicity, since amide spectral features are strongly related and sensitive to protein conformation. For each protein sample, both ATR-IR spectra of protein solution and of water solvent (constituting the background) have been collected. Fig. S12 shows the raw absorption spectrum of a protein in H_2O solution.

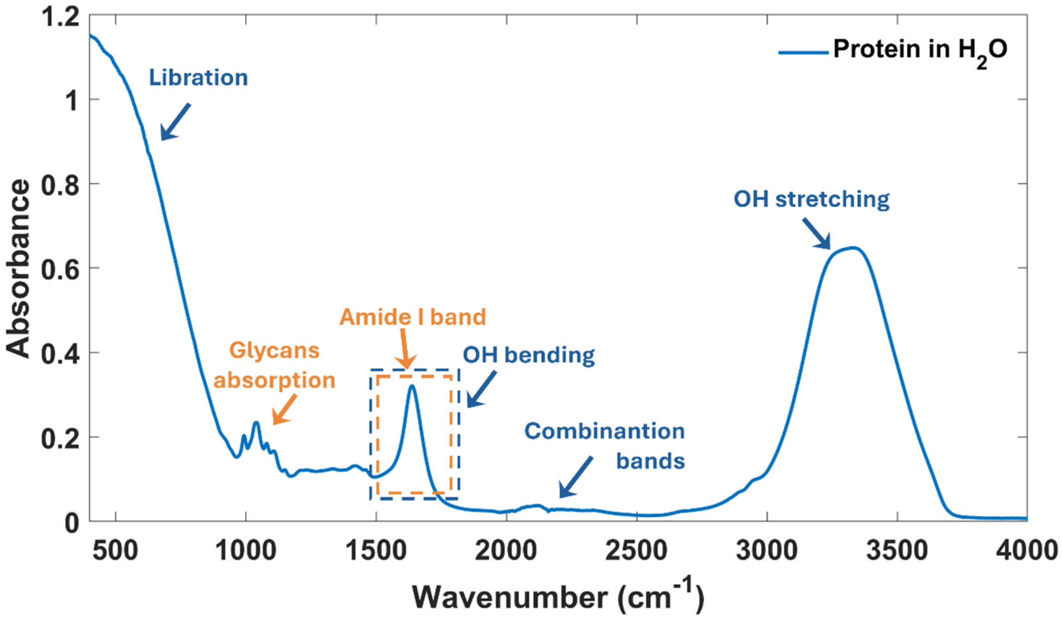

Figure S12. Raw absorption spectrum (blue curve) of SARS-CoV-2 S protein in H_2O solution. The contribution of H_2O vibrational modes to the overall absorption spectrum are indicated in cyan, consisting of water molecule libration at low frequency (around 500 cm^{-1}), OH bending around 1600 cm^{-1}, combination band between 2000-2500 cm^{-1} and OH stretching from 2800-3800 cm^{-1}. Absorption bands attributed to glycosylated viral protein are indicated in orange. They consist of the vibrations found between 900-1180 cm^{-1}, attributed to glycans, and the amide I band found between 1590-1720 cm^{-1} due to the amino acid C=O vibration.

First, a careful subtraction of the aqueous solvent was performed to correctly extrapolate the amide I signal from the overlapping absorption of H_2O bending mode. A routine was established for the proper subtraction of H_2O spectrum; then, spectral processing operations were applied (ATR correction, baseline correction, average, normalization) and the amide I band was then deconvoluted into its spectral components. The deconvolution was performed starting from the calculation of the 2nd-derivative absorption spectrum, whose minima establish the position in frequencies of convoluted absorption bands. A multiple gaussian fit was then performed with OPUS 8.2 software considering residual error (RMSE) value as goodness of fit parameter. While positions in frequency are fixed, the bandwidth and the peak intensity of convoluted components are left free. They are fitted evaluating RMSE value and paying attention to the proportionality between spectral components peak intensity and the one of respective 2nd-derivative minima. In Fig. S13 the amide I band of S protein is reported as an example with its 2nd-derivative, employed for the specific and unique identification of convoluted IR peak positions.

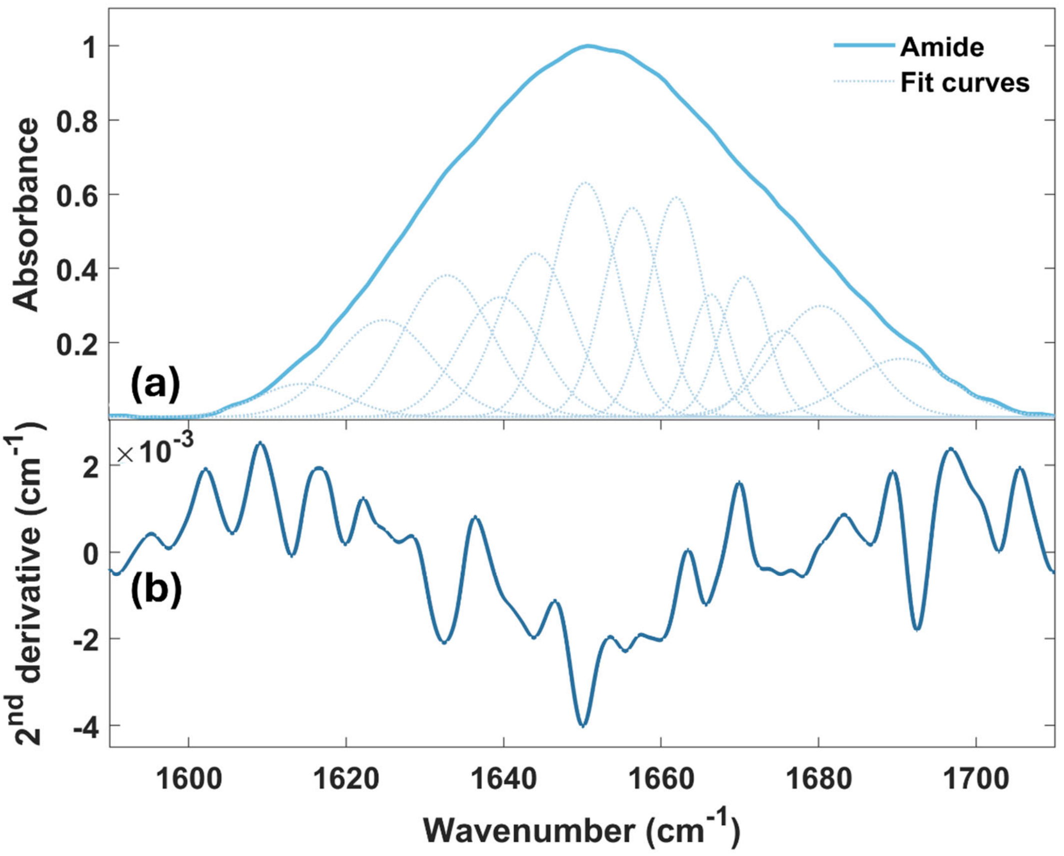

Figure S13. (a) IR amide I absorption band of S protein (thicker cyan curve), its deconvolution into spectral components (underlying dashed thinner curves) and (b) its 2nd-derivative (darker blue curve). Minima of 2nd-derivative corresponds to the position in frequency of amide spectral components.

Following the literature, each spectral component can be attributed to a secondary structure; the area of each convoluted band is then normalized to the total amide I integrated intensity, after the subtraction of side chain vibrations contribution. From this operation, the percentage value of each absorption band is calculated and related to the percentage estimation of the respective secondary structure content. The error associated with each secondary structure percentage content is calculated adapting the final fit of a protein on its different spectral measurement runs, calculating the standard deviation of each convoluted band integral and propagating this to the calculation of secondary structure percentage value.

The spectral analysis of CD spectra of proteins in H_2O solution is first performed the Jasco Spectra Manager™ software for basic spectra processing operations (background subtraction, average and unit conversion, see paragraph S2). The deconvolution is then performed employing CDPro Software, automatically comparing the experimental spectrum with properly built CD spectral database of already known proteins following specific algorithms. More specifically, CD spectra of S1 proteins have been analysed and deconvoluted in the wavelength range from 195 to 230 nm, referring to six different reference datasets, namely SP37, SP43, SDP42, SDP48, SMP50 and SMP56 basis sets, and employing three different algorithms, which are SELCON3, CDSSTR and CONTILL. The cross combination of these six different basis sets and these three algorithms lead to the CD spectra deconvolution in terms of secondary structure percentage content. Results are reported in following table.

Algorithm	Basis set	α				γ				ο			
		β-sheet	α-helix	β-turn	Random coil	β-sheet	α-helix	β-turn	Random coil	β-sheet	α-helix	β-turn	Random coil
SELCON3	SP37	41.29	6.46	21.91	30.34	33.27	13.73	20.02	32.98	39.61	8.18	20.39	31.82
	SP43	40.69	9.97	16.01	33.33	37.71	9.95	18.01	34.33	43.00	5.34	20.54	31.12
	SDP42	//	//	//	//	33.37	13.49	17.75	35.40	36.59	9.464	21.37	32.59
	SDP48	41.68	9.38	15.14	33.80	37.40	9.58	18.14	34.88	40.62	11.03	17.84	30.52
	SMP50	34.56	6.68	24.01	34.75	46.47	28.83	21.29	25.89	32.71	7.35	22.82	37.12
	SMP56	42.68	9.17	15.67	32.47	40.48	9.83	15.94	33.75	40.00	10.15	15.28	34.56
CDSSTR	SP37	48.98	5.89	24.49	20.63	35.09	16.45	22.33	26.12	43.10	6.70	24.50	25.70
	SP43	44.82	5.23	19.50	30.45	43.54	5.05	20.00	31.41	16.99	5.34	20.54	31.12
	SDP42	33.43	15.96	24.30	26.30	34.00	15.85	23.53	26.62	30.93	16.62	22.62	29.83
	SDP48	44.86	5.34	20.66	29.13	46.12	5.84	19.64	28.40	43.49	5.71	21.24	29.56
	SMP50	//	//	//	//	50.61	0.51	23.28	25.61	//	//	//	//
	SMP56	43.51	5.84	20.44	30.21	43.68	5.86	19.41	31.04	43.00	6.09	20.18	30.73
CONTINLL	SP37	24.30	0.40	27.90	47.40	29.23	19.02	21.72	30.03	32.50	0.70	26.80	40.00
	SP43	34.93	15.02	25.73	24.32	34.56	16.68	23.78	24.97	40.20	8.40	23.40	28.00
	SDP42	31.60	1.60	46.80	20.00	27.97	19.58	18.48	33.97	23.20	6.80	28.30	41.70
	SDP48	41.24	7.41	22.52	28.83	40.50	7.70	22.00	29.80	39.90	8.10	23.40	28.60
	SMP50	25.87	4.49	26.77	42.86	41.30	4.00	24.50	30.20	34.60	3.40	27.70	34.30
	SMP56	39.00	14.20	21.50	25.30	38.24	15.82	21.42	24.52	39.20	14.50	21.60	24.70

Figure S14. Secondary structure percentage contents of S1 proteins of α, γ and ο variants estimated from their CD spectra fitting performed through SELCON3, CDSSTR and CONTINLL algorithms employing SP37, SP43, SDP42, SDP48, SMP50 and SMP56 basis sets.

The final estimation of secondary structure percentage contents of variants S1 proteins is given as calculating the average and standard deviation of the results obtained using the cross combination of the six different basis sets and the three algorithms.
